# The enzymatic oxygen sensor cysteamine dioxygenase binds its protein substrates through their *N-*termini

**DOI:** 10.1016/j.jbc.2024.107653

**Published:** 2024-08-08

**Authors:** Karishma Patel, Yannasittha Jiramongkol, Alexander Norman, Joshua W.C. Maxwell, Biswaranjan Mohanty, Richard J. Payne, Kristina M. Cook, Mark D. White

**Affiliations:** 1 School of Chemistry, The University of Sydney, Camperdown, NSW, Australia; 2 School of Life and Environmental Sciences, The University of Sydney, Camperdown, NSW, Australia; 3 Faculty of Science, Charles Perkins Centre, The University of Sydney, Camperdown, NSW, Australia; 4 Australian Research Council Centre of Excellence for Innovations in Peptide and Protein Science, Sydney, NSW, Australia; 5 Sydney Analytical Core Research Facility, The University of Sydney, Camperdown, NSW, Australia; 6 Faculty of Medicine and Health, Charles Perkins Centre, The University of Sydney, Camperdown, NSW, Australia

**Keywords:** ADO, oxygen-sensing, hypoxia, protein degradation, posttranslational modification, *N*-degron pathway, enzyme kinetics, surface plasmon resonance, nuclear magnetic resonance

## Abstract

The non-heme iron-dependent dioxygenase 2-aminoethanethiol (aka cysteamine) dioxygenase (ADO) has recently been identified as an enzymatic oxygen sensor that coordinates cellular changes to hypoxia by regulating the stability of proteins bearing an *N*-terminal cysteine (*N*t-cys) through the *N*-degron pathway. It catalyzes O_2_-dependent *N*t-cys sulfinylation, which promotes proteasomal degradation of the target. Only a few ADO substrates have been verified, including regulators of G-protein signaling (RGS) 4 and 5, and the proinflammatory cytokine interleukin-32, all of which exhibit cell and/or tissue specific expression patterns. ADO, in contrast, is ubiquitously expressed, suggesting it can regulate the stability of additional *N*t-cys proteins in an O_2_-dependent manner. However, the role of individual chemical groups, active site metal, amino acid composition, and globular structure on protein substrate association remains elusive. To help identify new targets and examine the underlying biochemistry of the system, we conducted a series of biophysical experiments to investigate the binding requirements of established ADO substrates RGS5 and interleukin-32. We demonstrate, using surface plasmon response and enzyme assays, that a free, unmodified *N*t-thiol and *N*t-amine are vital for substrate engagement through active site metal coordination, with residues next to *N*t-cys moderately impacting association and catalytic efficiency. Additionally, we show, through ^1^H-^15^N heteronuclear single quantum coherence nuclear magnetic resonance titrations, that the globular portion of RGS5 has limited impact on ADO association, with interactions restricted to the *N*-terminus. This work establishes key features involved in ADO substrate binding, which will help identify new protein targets and, subsequently, elucidate its role in hypoxic adaptation.

Oxygen (O_2_) homeostasis is an essential biological process that regulates O_2_ supply and demand to avoid and alleviate hypoxic stress. At a molecular level, O_2_ concentrations are monitored by enzymatic O_2_ sensors which couple O_2_ availability to cellular activity through O_2_-dependent posttranslational modifications (PTMs). In animals, research in this area has historically focused on the hypoxia-inducible factor (HIF) system and its O_2_-sensing component prolyl hydroxylase domain, which coordinates whole scale transcriptional changes to low O_2_ (1, 2, 3). However, additional enzymes with the ability to sense and respond to physiologically relevant fluctuations in O_2_ concentration have recently been identified, including the non-heme iron-dependent dioxygenase 2-aminoethanethiol (cysteamine) dioxygenase (ADO) (4, 5, 6). ADO controls the stability of proteins bearing an *N*-terminal cysteine (*N*t-cys) in an O_2_-dependent manner through the *N*-degron pathway (4), which dictates the stability of a protein based on the identity and modification state of its *N*-terminal amino acid (7). *N*t-cys acts as a tertiary destabilising residue, requiring three sequential PTMs to promote proteasomal degradation of the protein, including oxidation, *N*t-arginylation, and ubiquitination. Under normoxic conditions, ADO catalyzes the first PTM, *N*t-cys sulfinylation, using both atoms of O_2_, which ultimately targets its protein substrate for removal (Fig. S1*A*) (4). However, during hypoxia, ADO is inactivated due to its high *K*_m_ and low affinity of O_2_, resulting in target stabilization and cellular change (4). Thus, ADO provides a direct link between O_2_ availability and protein levels.

ADO is a thiol dioxygenase (TDO) from the cupin fold superfamily of enzymes. In addition to regulating protein stability through the *N*-degron pathway, ADO also holds a prominent role in 2-aminoethanethiol (cysteamine) catabolism and taurine biosynthesis (8, 9). Taurine is an abundant nonproteogenic amino acid that is involved in many regulatory processes and the development of the central nervous system. As a part of this, ADO catalyzes cysteamine sulfinylation to produce hypotaurine, the precursor for taurine. Its active site sits in the center of a characteristic β-barrel structure, which is conserved in all TDOs (9, 10, 11). The catalytic center consists of a ferrous iron (Fe^2+^) that is octahedrally coordinated by a facial triad of three histidine residues, with three water molecules occupying the remaining ligation sites at rest (12, 13) (Fig. S1*B*). ADO retains low sequence and structural homology with cysteine dioxygenase, the only other mammalian TDO, which processes free cysteine as part of sulfur metabolism (8). ADO has a distinct distribution of amino acids in the active site, suggesting it uses divergent strategies to bind and modify its substrates. This is supported by various spectroscopic studies on the metal center, which imply that ADO coordinates its substrate in a monodentate (rather than bidentate) arrangement, although most of these experiments focused on cysteamine, the small molecule substrate of ADO (14, 15). ADO retains higher sequence, structural and functional homology with the plant cysteine oxidases (PCOs), which coordinate hypoxic adaptation in plants (16, 17, 18). The PCOs regulate the O_2_-dependent stability of various *N*t-cys proteins including group VII ethylene response factor, the polycomb repressive complex 2 subunit VERNALIZATION 2, and Little Zipper 2, all of which confer transcriptional changes to hypoxia, analogous to the HIF system in mammals (19, 20, 21, 22, 23). In contrast, established protein substrates of ADO include regulators of G-protein coupled signaling (RGS) 4, 5, and 16 (RGS4, RGS5, and RGS16) and the atypical cytokine interleukin 32 (IL32), which are predominantly associated with cell signaling events (4, 24), suggesting that ADO can complement the transcriptional output of HIF over a shorter timeframe in response to hypoxia (25). RGS4, RGS5, and RGS16 are implicated in maintaining healthy cardiovascular function and operate by binding G protein–coupled receptors subunits to enhance their GTPase activity, thereby abating G protein–coupled receptor–mediated responses in cells (24). IL32 on the other hand has been found to regulate proinflammatory cytokine networks and promote angiogenesis (26, 27). Furthermore, IL32 has been implicated in a range of pathologies, including infection, cancer, and autoimmune disease.

Although ADO is ubiquitously expressed, its protein substrates are limited to specific cell and/or tissue types (8). Over 200 *N*t-cys proteins are predicted to exist in humans, suggesting ADO can regulate the stability of additional *N*t-cys proteins in an O_2_-dependent manner. The RGS proteins, although related to each other, are distinct in function and composition to IL32, making it difficult to pinpoint sequence-based determinants of protein substrate engagement other than *N*t-cys (Fig. S1*C*). Furthermore, due to the lack of structural information regarding how ADO binds its targets, it remains unclear how ADO selects and interacts with its diverse repertoire of protein substrates.

To delineate the protein substrate binding requirements of ADO, we conducted a comprehensive study into the binding properties of established targets RGS5 and IL32 using surface plasmon resonance (SPR) and a LC-MS–based enzyme assay. We demonstrate that an unmodified substrate *N*t-thiol, an unmodified substrate *N*t-amino group and an active site iron are essential for binding and catalytic activity. Substituting the *N*t-thiol with a hydroxyl group could, however, mediate a reduced (∼150- to 160-fold) interaction with ADO. Furthermore, replacement of the iron cofactor with either zinc or cobalt preserved RGS5 and IL32 binding, albeit at the cost of enzymatic activity. Surprisingly, the remaining RGS5 and IL32 peptide sequence had a negligible impact on association, with the residues immediately next to *N*t-cys only moderately influencing binding and catalytic efficiency. Finally, ^15^N-heteronuclear single-quantum coherence (HSQC) nuclear magnetic resonance (NMR) experiments were conducted to gain a structural snapshot of the interaction between ADO and full-length protein, specifically RGS5. Titration of ADO into RGS5 induces chemical shift perturbations (CSPs) that are localized to the *N*-terminal region of RGS5 with the globular domain of the protein largely untouched. Together, these data establish the binding properties underpinning ADO protein substrate interactions for the very first time and determine the impact of chemical alterations, active site metal, sequence composition, and protein substrate tertiary structure on ADO association. Consequently, this work may provide a platform to aid the identification of novel ADO targets.

## Results

### ADO requires an unmodified *Nt-cys* for protein substrate binding

ADO catalyzes the O_2_-dependent sulfinylation of *N*t-cys residues, which reportedly bind to the iron cofactor in a monodentate arrangement through ligation of the thiol group (14, 15). To test the importance of *N*t-cys modifications and substitutions on ADO association, a SPR assay was established. Both kinetic and equilibrium analyses were performed, which displayed strong correlation. Data collected under steady state conditions is presented in the main text and kinetic constants are provided in the Tables S1–S8 (alongside steady state measurements). All measurements were collected at 4 °C to minimize enzyme activity.

In the first instance, peptides corresponding to the first 14 amino acids of the methionine excised *N* terminus of RGS5 and IL32 were generated through solid-phase peptide synthesis (SPPS), with and without alterations to the *N*t-cys (Fig. S1*C*). Biologically relevant modifications, including *N*t-acetylation (Ac-), *N*t-deamination (NH_2_ to H; deaminated-), and *N*t-sulfinylation (acquired through preincubation with ADO; -Ox), as well as mutations, including *N*t-cys to serine (-C2S) and *N*t-cys to alanine (-C2A), were chosen to evaluate the importance of each functional group on protein substrate association (chemical structures of the *N*t-cys modifications are shown in Tables S1 and S2).

**Figure 1:**
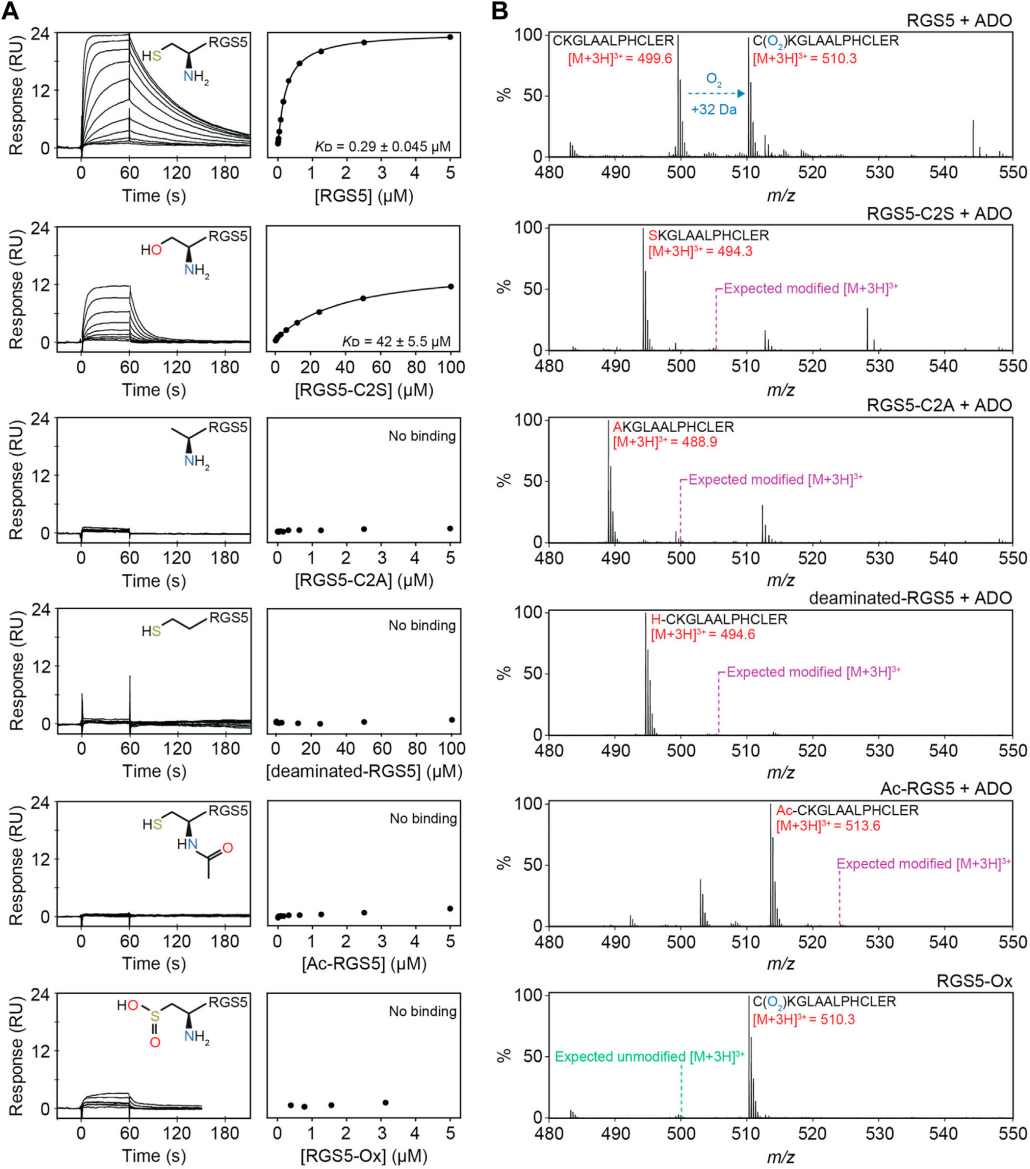
**ADO requires an unmodified *N*t-cys to interact with RGS5 peptides.***A*, *Left*: Representative SPR sensorgrams for the titrations of modified *N*t-cys RGS5 peptides with ADO. *Right*: fits of the equilibrium responses from the sensorgrams in the *left panels* to a 1:1 binding model. The identity, chemical structures of the modification state of the *N*t-cys of each peptide, and *K*ᴅ values are shown (*K*ᴅ given as the geometric mean of a minimum of three independent SPR measurements). *B*, LC-MS spectra showing the RGS5 peptide species detected following a 45 s incubation of the modified *N*t-cys RGS5 peptides (100 μM) with ADO (0.1 μM) at 37 °C (1-h incubations were also preformed, Fig. S4). The expected +32 da mass shift resulting from sulfinylation of the native RGS5 peptide in the presence of ADO is detected. The remaining RGS5 peptides do not get sulfinylated by ADO. The positions of the peptide species expected upon oxidation (or unmodified RGS5 in the case of the IL32-Ox peptide) by ADO are indicated. ADO, 2-aminoethanethiol dioxygenase; Nt-cys, *N*-terminal cysteine; SPR, surface plasmon resonance.

ADO could bind the native, unmodified RGS5 and IL32 peptides with submicromolar affinity, generating equilibrium dissociation constants (*K*ᴅ) of 0.29 ± 0.045 μM and 0.27 ± 0.005 μM, respectively (Fig. 1*A* and S2*A*). ADO could also bind the C2S substituents, but with significantly lower (∼150- to 160-fold) affinities of ∼40 μM, indicating that atomic O_2_ can only partially supersede the side chain sulfur (Figs. 1*A* and S2*A*). However, no binding was detected for the remaining *N*t-cys RGS5 and IL32 analogs, including the deaminated sequences, indicating that both an *N*t-thiol and *N*t-amino group are vital for protein substrate binding (Figs. 1*A* and S2*A*). Small increases in response units (RU) were observed for the titrations performed for RGS5-Ox and IL32-Ox that could be attributed to nonspecific contributions from the ADO used to prepare the sulfinylated peptide present in the samples (Fig. S3).

To confirm that *N*t-cys modifications and substitutions influence ADO activity, as well as binding, peptide turnover was monitored using an established LC-MS assay. In agreement with our SPR data, only the native RGS5 and IL32 peptide sequences could be oxidized by ADO (Figs. 1*B* and S2*B*). No ADO activity was detected with 100 μM modified *N*t-cys peptides following 1 h incubation at 37 °C, including *N*t-acetylated and *N*t-deaminated peptides, which retain an enzymatically active thiol group (Fig. S4, *A* and *B*). Together, this indicates that a free, unmodified *N*t-thiol and *N*t-amine are required for ADO protein substrate binding and turnover.

### ADO requires an active site metal for protein substrate binding

Spectroscopic studies have indicated that the non-heme iron cofactor of ADO coordinates the thiol group of the RGS5 *N*t-cys (14, 15). To investigate the importance of this ligation in terms of protein substrate binding, we measured the association of native RGS5 and IL32 peptide with immobilized ADO following metal chelation and reconstitution on chip using the SPR assay described above. Treatment with a mixture of 1, 10-phenanthroline (10 mM) and EDTA (100 mM) removed almost all the protein-bound iron from ADO in solution, as indicated by inductively coupled plasma (ICP)-mass spectrometry (MS) elemental analysis (Fig. S5*A*).

**Figure 2:**
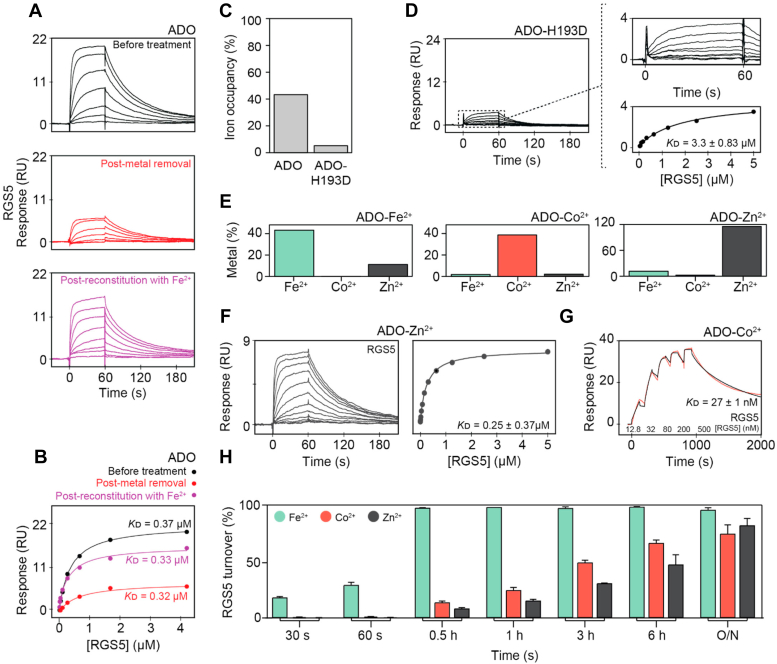
**ADO requires an active site metal for substrate binding.***A*, Representative SPR sensorgrams for the titration of the native RGS5 peptide with ADO before treatment (*top*), postmetal removal using 10 mM 1, 10-phenanthroline and 100 mM EDTA (*middle*), and postreconstitution with iron using 0.1 mM FeSO_4_ (supplemented with 12.5 mM sodium ascorbate) (*bottom*). *B*, Fits of the equilibrium responses from the sensorgrams in (*A*) to a 1:1 binding model. The *K*ᴅ values presented are calculated from the specific SPR data presented in this figure. *C*, the iron occupancy of the native ADO and ADO-H193D determined by ICP-MS. The occupancy is provided as a percent based on amount of iron detected in the sample relative to the concentration of ADO present. *D*, *Left*: Representative SPR sensorgram for the titration of native RGS5 peptide with ADO-H193. *Right*: Fits of the equilibrium responses from the sensorgrams in the *left panels* to a 1:1 binding model. The *K*ᴅ value is shown (*K*ᴅ given as the geometric mean of a minimum of three independent SPR measurements). *E*, The metal (Fe^2+^, Co^2^, and Zn^2+^) occupancies of native ADO (*left*), ADO-Co^2+^ (*middle*), and ADO-Zn^2+^ (*right*) determined by ICP-MS. The occupancies are provided as percentages based on amount of the metals detected in the sample relative to the concentration of ADO present. *F*, *Left*: Representative SPR sensorgram for the titration of the native RGS5 peptide with ADO-Zn^2+^. *Right*: Fits of the equilibrium responses from the sensorgrams in the *left panels* to a 1:1 binding model. The *K*ᴅ value is shown (*K*ᴅ given as the geometric mean of a minimum of three independent SPR measurements). *G*, Representative single cycle kinetic (SCK) SPR sensorgram of the native RGS5 peptide with ADO-Co^2+^. The sensorgram is shown in *red* and the fit to the data is shown in *black*. The concentrations of RGS5 used in the titration and the *K*ᴅ value is shown (*K*ᴅ given as the geometric mean of a minimum of three independent SPR measurements). *H*, Time course showing the enzymatic turnover of the RGS5 peptide (100 μM) by native ADO, ADO-Co^2+^, and ADO-Zn^2+^ (0.1 μM enzyme) at 37 °C. The average of three independent experiments are shown (error bars show the standard error). O/N indicates overnight. ADO, 2-aminoethanethiol dioxygenase; ICP, inductively coupled plasma; MS, mass spectrometry; Nt-cys, *N*-terminal cysteine; SPR, surface plasmon resonance.

Prior to treatment, a saturated binding profile with a maximum response (R_max_) of ∼20 RU could be detected for native RGS5 and IL32 peptides with ADO (Fig. 2, *A* and *B*, and S5, *B* and *C*). However, a significant loss in R_max_ (∼80%) was observed following chelation of the immobilized ADO-bound metal (Fig. 2, *A* and *B*, and S5, *B* and *C*). This reduction, which did not impact binding affinity, indicates a decrease in the active population of ADO on the SPR surface, as R_max_ is directly proportional to the quantity of functional immobilized sample.

To verify if the depleted signal is caused by metal chelation, the immobilized 1, 10-phenanthroline and EDTA-treated ADO was washed and reconstituted with FeSO_4_ (0.1 mM supplemented with 12.5 mM sodium ascorbate) before being reassessed for peptide association. A noticeable increase in R_max_, comparable to the untreated ADO, was observed, confirming that iron can rescue binding and, additionally, indicating that metal coordination is essential for ADO protein substrate association (Fig. 2, *A* and *B*, and S5, *B* and *C*).

To further validate the importance of metal coordination in protein substrate binding, an ADO-H193D mutant, which is known to impair iron retention, was generated (Fig. S1*B*). A significant reduction in metal occupancy was confirmed by ICP-MS (Fig. 2*C*). This correlated with a low SPR-binding response of approximately ∼2 to 3 RU, indicating a reduced population of substrate-binding ADO on the chip relative to the total amount of immobilized ADO (Figs. 2*D* and S5*D*). Furthermore, the calculated *K*ᴅ values with native RGS5 and IL32 peptides were ∼13-fold and ∼77-fold weaker, respectively, than the interactions with WT ADO (Table S3). 1D ^1^H-NMR analysis of WT ADO and ADO-H193D shows that both proteins possess the same general fold, suggesting that this decrease is due to metal loss, not global changes in structure (Fig. S5*E*).

To study if different metals can compensate for the inherent iron cofactor, zinc (Zn^2+^) and cobalt (Co^2+^) substituted ADO was produced through minimal media expression, validated by ICP-MS, and analyzed for binding by SPR (Fig. 2*E*). ADO-Zn^2+^ could bind both native RGS5 and IL32 peptides with similar affinities to iron-incorporated ADO (Figs. 2*F* and S5*F*). However, ADO-Co^2+^ exhibited a ∼10-fold increase in binding affinity with a significantly slower off-rate relative to native ADO, indicating robust and sustained association of the RGS5 and IL32 peptides with the enzyme (Figs. 2*G* and S5*G*, Table S3).

The catalytic functionality of the metal substituted ADO proteins was subsequently assessed by LC-MS using RGS5 peptide as a representative protein substrate. Both ADO-Zn^2+^ and ADO-Co^2+^ had significantly impaired activity relative to iron bound ADO, exhibiting no turnover over short time periods (Fig. 2*H*). However, product formation was observed following long incubation times (≥30 min), with ∼80% conversion detected overnight, which is likely attributed to the activity of residual iron bound ADO (Fig. 2, *E* and *H*). Together, these findings indicate that different transition metals can substitute iron to facilitate protein substrate binding but not catalysis.

### Residues immediately next to *Nt-cys* influence protein substrate binding

Having established that an unmodified *N*t-cys and active site metal are vital for ADO protein substrate binding, we conducted an alanine scan to determine the role of downstream substrate residues on association. Peptides corresponding to the first 14 amino acids of the methionine-excised *N-*terminus of RGS5 and IL32 were generated by SPPS, with alanine systematically substituting residues three to 15 (with the initiating methionine denoted as 1) and measured for binding using the SPR assay established in this study (Figs. S6 and S7, Tables S4 and S5).

**Figure 3:**
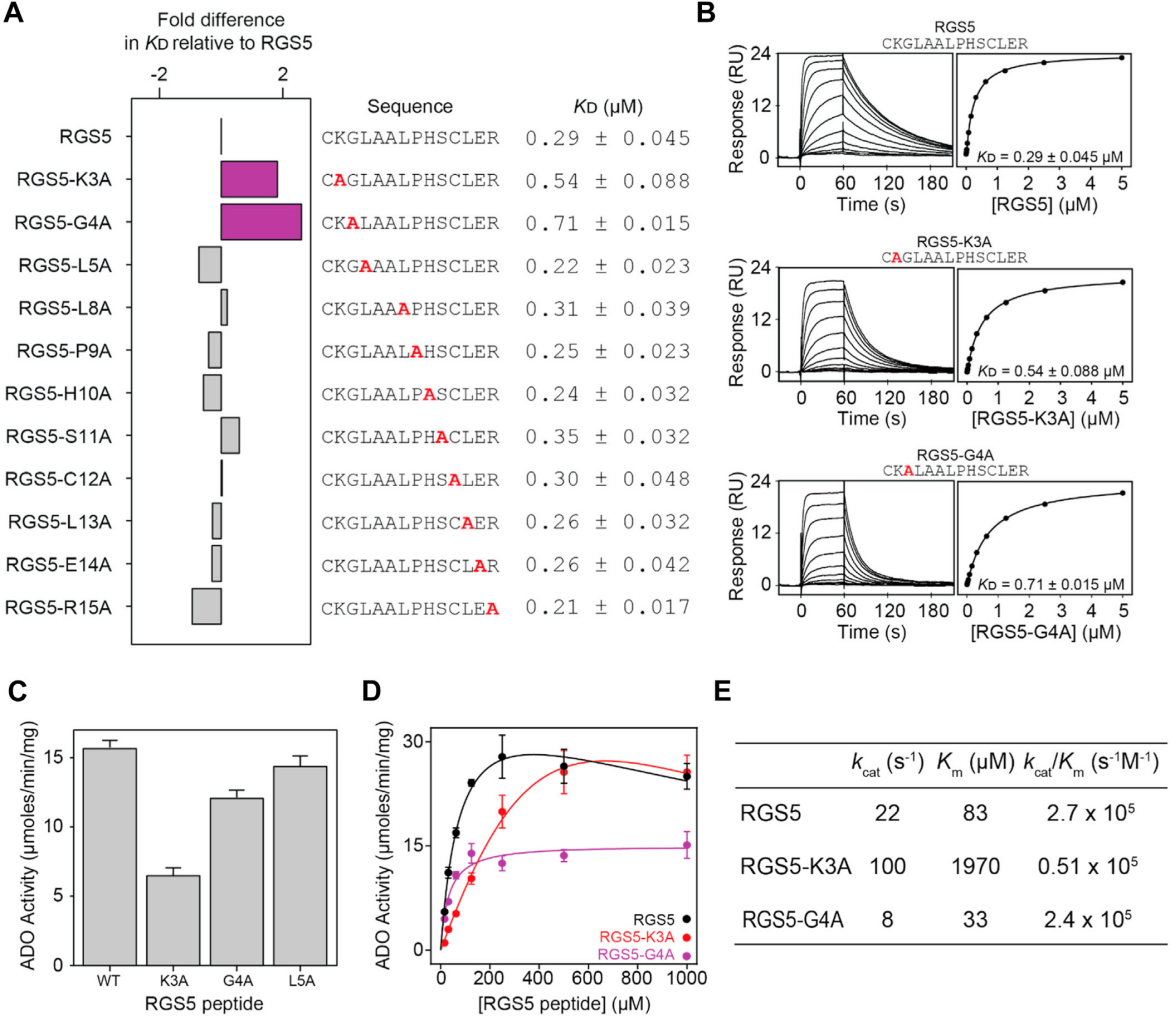
**SPR and enzyme kinetic analyses of the RGS5 alanine mutational scan.***A*, Fold difference in *K*ᴅ relative to the native RGS5 peptide. The substitutions that generate a greater than 1.5-fold reduction in *K*ᴅ are colored in *purple*. The peptide sequences, with the mutation indicated in *bold red* lettering, and *K*ᴅ values for the interactions with ADO are provided (*K*ᴅ given as the geometric mean of a minimum of three independent SPR measurements). *B*, *Left*: Representative SPR sensorgram for the titration of native RGS5, RGS5-K3A, and RGS5-G4A with ADO. *Right*: Fits of the equilibrium responses from the sensorgrams in the *left panels* to a 1:1 binding model. The *K*ᴅ value is shown (*K*ᴅ given as the geometric mean of a minimum of three independent SPR measurements). Data for the native RGS5 peptide are provided for reference (first presented in Fig. 1*A*). *C*, The specific activity of ADO (0.05 μM) calculated by measuring the rate of native RGS5, RGS5-K3A, RGS5-G4A, and RGS5-L5A oxidation by LC-MS (100 μM peptide, 45 s incubation at 37 °C). *D*, Michaelis–Menten kinetic plots for native RGS5, RGS5-K3A, and RGS5-G4A performed in aerobic conditions at 37 °C. The average of three independent experiments are shown (error bars show the standard error). *E*, Table of the reaction kinetics for ADO catalysis of native RGS5, RGS5-K3A, and RGS5-G4A calculated from the data presented in D. ADO, 2-aminoethanethiol dioxygenase; SPR, surface plasmon resonance.

Relatively moderate changes in *K*ᴅ were observed for the RGS5 alanine substituted peptides, with RGS5-K3A and RGS5-G4A (the residues immediately next to *N*t-cys) having the most noticeable impact on binding (Fig. 3*A*). These substitutions decreased the binding affinity ∼2-fold and ∼2.5-fold relative to the native sequence (0.29 ± 0.045 μM), producing *K*ᴅ values of 0.54 ± 0.088 and 0.71 ± 0.015 μM, respectively (Fig. 3, *A* and *B*). A more significant change in dissociation was observed for IL32, with IL32-F3A (the residue next to *N*t-Cys) generating a *K*ᴅ of 2.2 ± 0.072 μM, which is ∼8-fold weaker than the native sequence (0.27 ± 0.005 μM) (Fig. S8, *A* and *B*). IL32-L7A also reduced binding affinity, producing a *K*ᴅ of 0.57 ± 0.012 μM (Figs. S7 and S8*A*). Interestingly, the substitutions IL32-P4A and IL32-K5A improved binding affinity approximately 2-fold, generating *K*ᴅ values of 0.15 ± 0.0012 and 0.14 ± 0.0020 μM, respectively (Fig. S8, *A* and *B*). Taken together, this suggests that the substrate residues immediately next to *N*t-cys can influence binding, with amino acids possessing larger side chains preferred in position three and amino acids with conformational flexibility preferred in position four.

To determine how the two substrate residues immediately next to *N*t-cys influence ADO activity, a stopped LC-MS assay was employed to calculate and compare kinetic parameters using the Michaelis–Menten enzyme model. These experiments were conducted at 37 °C under atmospheric O_2_ concentrations. The native RGS5 peptide generated a turnover number (*k*_cat_) and Michaelis constant (*K*_m_) of 22 s^−1^ and 83 μM, respectively, which are comparable to previously published values (Fig. 3, *D* and *E*) (4). The RGS5-K3A substitution produced a *k*_cat_ of 100 s^−1^ and a *K*_m_ of 1970 μM, while the RGS5-G4A peptide generated a *k*_cat_ of 8 s^−1^ and a *K*_m_ of 33 μM, parameters that are largely different from the native RGS5 peptide, indicating that the substitutions can influence both binding and turnover.

The native IL32 peptide generated a *k*_cat_ of 8 s^−1^ and a *K*_m_ of 24 μM (Fig. S8, *D* and *E*). The IL32-F3A peptide produced a *k*_cat_ of 9 s^−1^, which is comparable to the native peptide, but a higher *K*_m_ of 88 μM, indicating that the substitution reduces affinity but does not alter catalysis. The IL32-P4A peptide also generated a *k*_cat_ comparable to the native IL32 peptide (7 s^−1^) but a reduced *K*_m_ of 19 μM, suggesting that ADO has greater affinity for the IL32-P4A peptide. Together, these data highlight how protein substrate composition can affect different kinetic parameters.

To sequentially test the importance of the second and third position amino acids in mediating an interaction with ADO, we measured the binding of di-RGS5 and tri-RGS5 and IL32 peptides to ADO (Table S6). ADO bound the RGS5 and IL32 dipeptides with 1.2 ± 0.22 μM and 0.91 ± 0.094 μM affinity, respectively, ∼4-fold weaker than the 14-amino acid long native peptides used elsewhere in this study (Fig. S9, *A* and *B*). ADO bound the RGS5 tripeptide with 0.46 ± 0.024 μM affinity, only ∼1.5-fold weaker than the 14 residue RGS5 peptide, confirming that the first three residues of RGS5 confer the greatest contributions to protein substrate binding (Fig. S9, *C* and *E*). Surprisingly, ADO bound the IL32 tripeptide with an affinity of 0.13 ± 0.0037 μM, which is ∼2-fold stronger than the representative 14 residue long IL32 peptide, indicating that the first three residues of IL32 confer the greatest contribution to protein substrate binding, with downstream residues negatively impacting association (Fig. S9, *D* and *F*).

Finally, we selected RGS5 to probe the influence of residues with different chemical properties at position three and, to a lesser extent, four. A range of amino acids with varying steric bulk and charge were sampled at position three, while two mutants, G4P (to mimic the sequence of IL32) and G4K (to introduce size and charge), were generated at position four (Table S7). All substitutions compromised binding relative to the native peptide sequence, with large (including K3Y and K3W), nonpolar (K3L), and positively charged (K3R) residues at position three the most tolerated (Fig. S10). However, K3D and G4K had the most detrimental effect on binding, generating *K*_D_ values of 39 ± 1.2 and 12 ± 1.5 μM, respectively, which corresponds to a 135- and 41-fold decrease in association relative to the native peptide sequence (Fig. S10*A*). The turnover of these sequences was subsequently analyzed, revealing reduced activity over short time periods, particularly for K3D, which was oxidized approximately 8 times slower than the native peptide (Fig. S10*B*). However, over 45% conversion was observed for all variants following 1 h incubation, suggesting that the amino acids immediately next to Nt-cys can influence, but not necessarily prevent, ADO binding and turnover (Fig. S10*B*).

### Kinetic and binding studies on full-length RGS5

All reported studies on recombinant ADO (at least in terms of the *N*-degron pathway) have used peptides as representative protein substrate. To determine the influence of full-length protein and tertiary structure on substrate binding and ADO activity, we expressed and purified full-length human RGS5.

**Figure 4:**
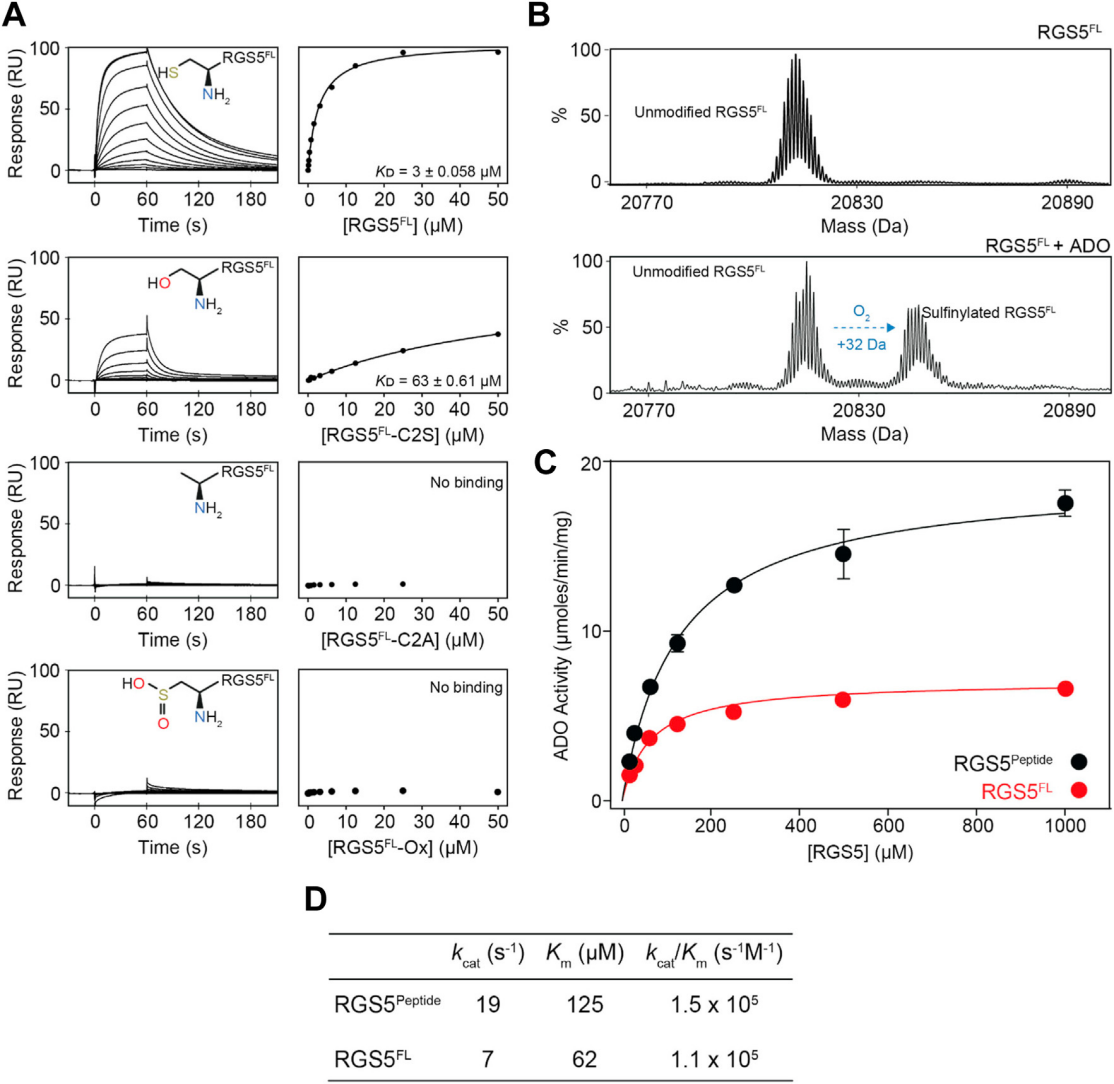
**SPR binding and kinetic studies of the interaction between ADO and full-length RGS5.***A*, *Left*: Representative SPR sensorgrams for the titrations of modified *N*t-cys RGS5 full-length proteins with ADO. *Right*: Fits of the equilibrium responses from the sensorgrams in the *left panels* to a 1:1 binding model. The identity, chemical structures of the modification state of the *N*t-cys of each protein, and *K*ᴅ values are shown (*K*ᴅ given as the geometric mean of a minimum of three independent SPR measurements). *B*, LC-MS spectra showing the RGS5 protein species detected following a 45 s incubation of full-length RGS5 (100 μM) with and without ADO (0.1 μM) at 25 °C. The expected +32 Da mass shift resulting from sulfinylation of RGS5 in the presence of ADO is detected. *C*, Michaelis–Menten kinetic plots for native RGS5 peptide (RGS5^Peptide^) and full-length protein (RGS5^FL^) performed in aerobic conditions at 25 °C. The average of three independent experiments are shown (error bars show the standard error). *D*, Table of the reaction kinetics for ADO catalysis of RGS5^Peptide^ and RGS5^FL^ calculated from the data presented in (*C*). ADO, 2-aminoethanethiol dioxygenase; Nt-cys, *N*-terminal cysteine; SPR, surface plasmon resonance.

Interactions between ADO and full-length RGS5 (RGS5^FL^) were initially examined by SPR, as described above, revealing a *K*ᴅ of 3 ± 0.058 μM (Fig. 4*A*). This is ∼10-fold higher than RGS5 peptide, suggesting that globular protein has a negative impact on binding affinity. A RGS5^FL^-C2S mutant increased the *K*ᴅ to 63 ± 0.61 μM, while both preincubation of RGS5^FL^ with ADO (to promote *N*t-cys sulfinylation) and RGS5^FL^-C2A prevented binding, as observed for RGS5 peptide, again emphasizing the vital role the *N*t-cys plays in protein substrate association (Fig. 4*A*). Furthermore, metal chelation analysis for RGS5^FL^ mirrored the RGS5 peptide results, where treatment with 10 mM 1, 10-phenanthroline and 100 mM EDTA drastically decreased R_max_ with no major impact on *K*ᴅ, indicating a reduction of active ADO on the SPR surface, which could be recovered through reconstitution of iron, highlighting the importance of metal coordination in facilitating protein substrate binding (Fig. S11, *A* and *B*). The RGS5^FL^-C2S mutant displayed the same pattern of R_max_ reduction and rescue following metal chelation and iron reconstitution confirming that the hydroxyl group of *N*t-ser likely coordinates the iron cofactor in a similar manner to *N*t-thiol (Fig. S11, *C* and *D*).

To determine whether variation in binding affinity correlates with enzyme activity, a stopped LC-MS assay was employed to monitor the initial rate of reaction at different substrate concentrations so that kinetic parameters of RGS5 peptide and RGS5^FL^ protein can be calculated and compared using the Michaelis–Menten model of enzyme kinetics (Fig. 4, *B* and *C*). As incubation at 37 °C caused RGS5^FL^ to precipitate, these experiments were conducted at 25 °C under atmospheric O_2_ concentrations. Surprisingly, a reduction in both *k*_cat_ and *K*_m_ was observed for full-length RGS5 relative to peptide (Fig. 4, *C* and *D*), suggesting that the tertiary structure of RGS5^FL^ decreases turnover and increases affinity.

### NMR studies of the interaction between ADO and full-length RGS5

To structurally delineate the binding mode of full-length RGS5, we performed ^15^N-HSQC CSP experiments for RGS5 in the absence and presence of ADO. Based on observations that the substitution C2S facilitates binding, albeit relatively weakly, without turnover and product dissociation, this mutant was selected for initial NMR spectroscopy experiments. A uniformly ^15^N- and ^13^C-labeled RGS5^FL^-C2S was produced and used to make sequence-specific chemical shift assignments (H^N^, N, ^13^C^α^, and ^13^C^β^) in isolation using standard triple resonance approaches (Fig. S12, Biomagnetic Resonance Bank entry: 52267). A set of well dispersed peaks corresponding to the folded RGS domain of the protein and a group of dense overlapping peaks that could be assigned to the *N*-terminal portion of RGS5^FL^-C2S were observed (Figs. S12 and S13*A*). Secondary structure analysis of the protein performed using the 3D NMR spectra collected to make backbone assignments confirmed that the *N*-terminal tail of RGS5^FL^-C2S is disordered in solution (Fig. S12*B* and Table S8).

**Figure 5:**
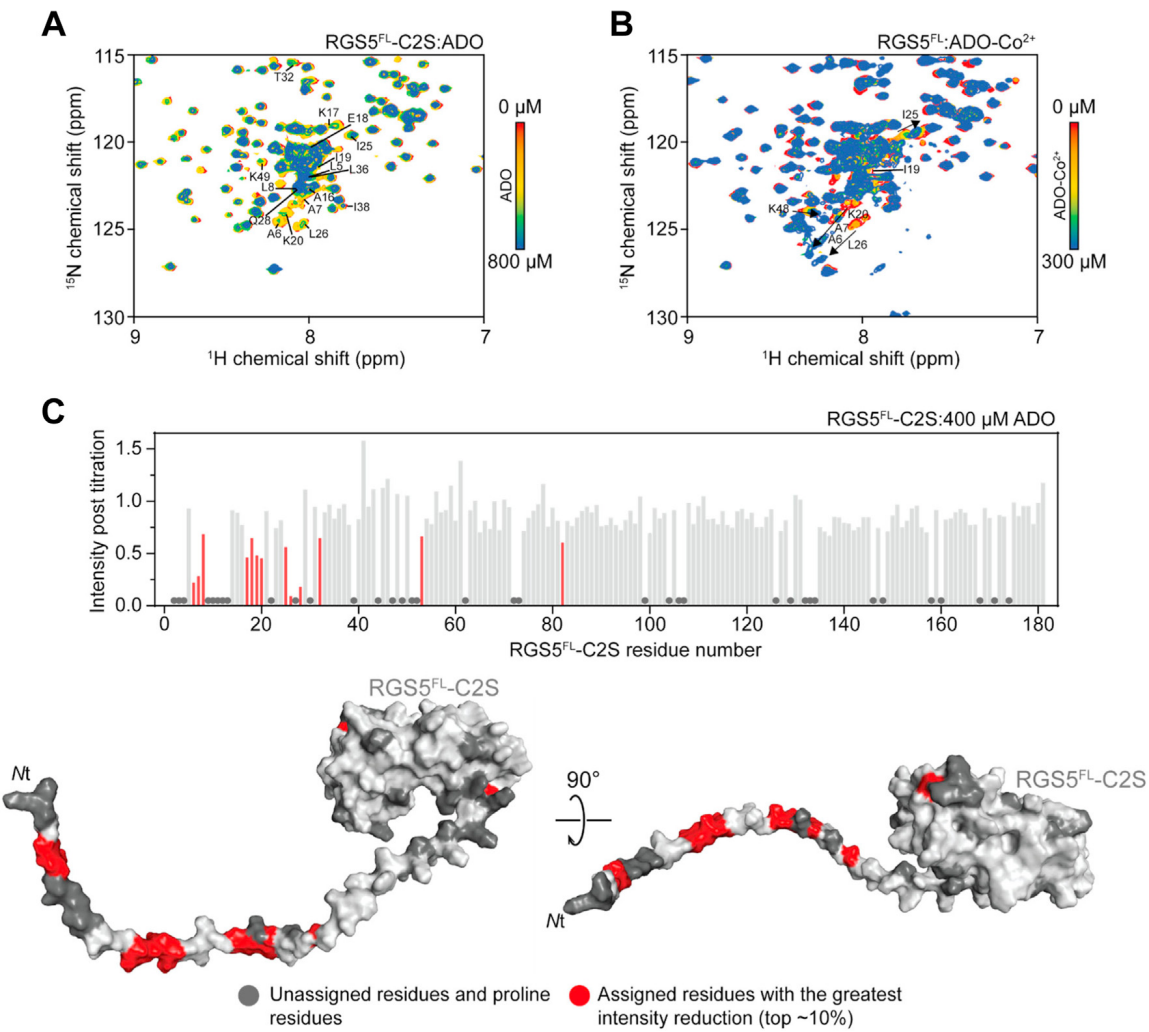
**NMR studies of the interaction between ADO and full-length RGS5.** For all titrations, RGS5 was used at a concentration of 50 μM and the assignments for some signals are indicated. *A*, ^15^N-HSQC spectra of RGS5^FL^-C2S alone (*red*) and in the presence of increasing concentrations of ADO (up to 800 μM, *blue*). *B*, ^15^N-HSQC spectra of RGS5^FL^ alone (*red*) and in the presence of increasing concentrations of ADO-Co^2+^ (up to 300 μM, *blue*). The directions of chemical shift change for selected peaks are indicated by *arrows*. *C*, T*op*: Quantitation of signal intensity post titration of 400 μM ADO into RGS5^FL^-C2S. *Bottom*: The residues that display the greatest reduction in signal intensity (∼*top* 10%) are mapped onto the AlphaFold model of RGS5 (*gray*) in *red*. Unassigned residues and proline residues (which do not have signals in ^15^N-HSQC spectra) are depicted in *dark gray*. ADO, 2-aminoethanethiol dioxygenase; HSQC, heteronuclear single-quantum coherence; Nt-cys, *N*-terminal cysteine; SPR, surface plasmon resonance.

Titration of WT ADO into the uniformly ^15^N-labeled RGS5^FL^-C2S induced CSPs in the portion of the ^15^N-HSQC corresponding to the disordered *N* terminus of RGS5, which is indicative of a specific interaction between this portion of the protein and ADO (Fig. 5*A*). Increasing concentrations of ADO caused a decrease in the intensity of these peaks, although no new peaks were observed. This selective reduction in signal intensity pattern indicates that the interaction is in intermediate exchange on the chemical shift timescale, consistent with the micromolar binding affinity measured by SPR. With increasing concentrations of ADO, a global reduction in peak intensity was also observed that is likely attributed peak broadening as a result of RGS5^FL^-C2S:ADO complex (a protein complex that is relatively large and favors fast transverse relaxation time) (Fig. 5*A*).

To confirm this interaction reflects the binding interactions of a native *N*t-cys, WT RGS5^FL^ was produced and assigned, which generated near identical spectra to RGS5^FL^-C2S (Fig. S14). Cobalt-substituted ADO (ADO-Co^2+^), which is catalytically impaired (especially over the lifetime of NMR data collection), was titrated into ^15^N-labeled RGS5^FL^ to capture native binding interactions. As observed for iron bound ADO and RGS5^FL^-C2S, CSPs were isolated to peaks corresponding to the *N*-terminal region of RGS5^FL^ (Fig. 5*B*). The CSPs displayed signatures of a slow-to-intermediate exchange regime, with both the gradual disappearance and appearance of new peaks observed alongside peak broadening. These observations are consistent with the stronger binding that ADO-Co^2+^ displays for its protein substrates relative to native iron-bound ADO.

To verify if the RGS5^FL^ and RGS5^FL^-C2S CPSs represent an ADO binding event, iron-bound ADO was titrated into RGS5^FL^ to promote *N*t-cys sulfinylation, which inhibits association as demonstrated by SPR. No significant differences in the RGS5^FL^ HSQC before and after an overnight incubation with ADO were observed, indicating no interaction (Fig. S15*A*). The fact that no major CSPs were observed after RGS5^FL^ sulfinylation supports previous observations that the globular portion of RGS5 does not participate in the ADO–RGS5 interaction. Furthermore, when the metal deficient ADO-H193D mutant, described earlier, was titrated in RGS5^FL^-C2S, no interaction was observed, confirming that an active site metal is critical for protein substrate binding. A few selected peaks that do show minor CSPs in the ADO:RGS5^FL^ and ADO-H193:RGS5^FL^-C2S titrations are also shifted in the overlaid RGS5^FL^ and RGS5^FL^-C2S spectra (Fig. S14), suggesting that these residues are likely sensitive to small pH differences due to buffer mismatch between samples.

Finally, we sought to map the intensity changes observed in our titrations onto an AlphaFold model of RGS5. The spectra for the titration of 400 μM ADO into RGS5^FL^-C2S was selected for the analysis due to the clear quality of the spectra and a lack of global peak disappearance (Fig. S15*C*). The peaks that showed the greatest reduction in intensity correspond to amino acids found at the very *N* terminus of the ∼40 residue long disordered stretch in RGS5 supporting our findings that the globular portion of RGS5 is not necessary for binding.

Overall, this data indicates that an unmodified *N*t-Cys residue and active site metal are critical for ADO substrate binding, with surprisingly little contributions made from the remainder of the protein.

## Discussion

The non-heme iron-dependent dioxygenase ADO has recently been identified as an enzymatic O_2_ sensor that coordinates cellular changes to hypoxia by regulating the stability of *N*t-cys proteins through the *N*-degron pathway. ADO is ubiquitously expressed, however, a limited number of cell and/or tissue type specific protein substrates have been verified, including RGS4, RGS5, and IL32, suggesting that ADO can process additional *N*t-cys proteins in an O_2_-dependent manner. To help ascertain new targets and explore the underlying biochemistry of the enzyme, we conducted a substrate binding study to establish the features required for ADO association and activity.

Initial experiments analyzing *N*t-cys modifications and substitutions indicated that a free thiol and amine group are vital for protein substrate binding and turnover. Removal or modification of either group impaired both ADO association and activity, revealing that the enzyme has high selectivity for an unmodified *N*t-Cys. Only the substitution C2S retained some, albeit weaker, affinity for ADO, rendering it an amenable protein substrate analog for subsequent structural characterization by NMR. Similar strategies where the thiol is replaced with a hydroxyl to avoid oxidation and maintain an extended interaction have been employed for other TDOs (28). Interestingly, *N*-terminal acetylation prevented both binding to, and turnover by, ADO, suggesting that acetylation may play a role in regulating the stability of *N*t-cys proteins through the *N*-degron pathway *in vivo*. Small *N*-terminal amino acid residues like cysteine can be acetylated by the acetyltransferase complex NatA in mammals (29, 30). It is possible that NatA increases the stability of *N*t-cys substrates by impairing association with ADO. This would add an extra level of control and complexity to the Cys/Arg branch of the *N*-degron pathway. It would also complicate the identification of novel ADO targets through traditional knockdown/overexpression experiments. Additional measures restricting or reversing *N*-terminal acetylation may need to be considered to find and/or verify new ADO protein substrates. An investigation exploring this concept was published during revision of this manuscript, revealing that ADO and NatA have contrasting substrate sequence preferences, which may accentuate what *N*t-cys proteins, are regulated in an O_2_-dependent manner through the *N*-degron pathway (31).

The role of the iron cofactor in protein substrate binding was explored through SPR on-chip metal chelation and site directed mutagenesis, revealing that association is proportional to metal occupancy. Taken together with the *N*t-cys modification and substitution data, this suggests that metal coordination is a fundamental interaction which is critical for ADO protein substrate binding, as enzyme structure appears unaltered by metal extraction. Spectroscopic studies on ADO propose that the substrate thiol ligates the iron cofactor in a monodentate arrangement, leaving two coordination sites for O_2_ to bind in a side on orientation (14, 15, 32). However, most of these experiments were conducted with cysteamine, the small molecule substrate of ADO, used nitric oxide as a spin surrogate, which could impede coordination, or employed ferric iron, which is not catalytically active. Our results indicate that a free *N*-terminal amine is vital for peptide/protein association. Although the amine might participate in a protein-mediated interaction, such as one with D206 (human numbering) which is strategically positioned in the active site of ADO (12, 13) and important for PCO activity (16), additional studies, including structural, are required to confirm its role and exclude bidentate coordination, which is frequently reported for other TDOs including the homolog cysteine dioxygenase (33).

Interestingly, alternative transition metals could substitute iron in terms of protein substrate binding, with cobalt enhancing association approximately 10-fold, which may facilitate elucidation of a substrate bound X-ray crystal structure. However, trace iron is always retained during purification, suggesting that ADO coordinates its cofactor tightly, in contrast to other non-heme iron-dependent dioxygenase such as prolyl hydroxylase domain, which often requires exogenous iron to maintain full activity *in vitro* (34, 35). This explains the slow turnover observed with metal substituted enzyme and correlates with *in cellulo* studies where chemical chelation cannot totally ablate ADO activity (4, 25).

An alanine scan of peptides corresponding to known protein substrate sequences demonstrated that ADO could tolerate substitutions in every position downstream of Nt-Cys. However, the residues next to *N*t-cys caused the most notable changes in binding and activity, indicating that they have the biggest influence on protein substrate association and turnover. This is particularly true for K3 in RGS5 and F3 in IL32, the residues immediately adjacent to *Nt*-Cys. Furthermore, ADO appears to prefer flexibility in position four as removal of a fortuitously located glycine (RGS5) and proline (IL32) reduced and increased binding and activity, respectively. Investigation into whether ADO can accommodate amino acids with different chemical compositions at the third and fourth positions of RGS5 peptides found that all of the modifications trialed to bind ADO, albeit with moderately reduced binding relative to the native sequence. A negative charge at the third position (RGS5-K3D mutation) and a bulky residue at the fourth position (RGS5-G4K) had the most detrimental effect on both binding and enzymatic activity (although both were turned over to a relatively high level following long incubation periods), whereas positively charged (arginine) and aromatic (tyrosine and tryptophan) residues were the best tolerated. These findings reflect those from a study published during revision of this manuscript (31). Nevertheless, preliminary screening of potential protein substrate sequences indicates that ADO can process a wide range of peptide compositions, albeit with different activities. This is perhaps unsurprising as mammals only encode one *N*t-cys oxidase, in contrast to plants which express multiple isoforms (4, 36). Accordingly, ADO may be promiscuous in nature so it can regulate the stability of a diverse pool of *N*t-cys substrates.

Although there does not appear to be a reliable relationship between dissociation constants obtained by SPR and individual kinetic parameters calculated through enzyme assays (with changes in *k*_cat_ and/or *K*_m_ observed), there is a consistent correlation with catalytic efficiency as determined through *k*_cat_/*K*_m_. This suggests that ADO turnover can contribute to SPR measurements, even though various steps were taken to exclude, or at least limit the impact of, product formation on results, including maintaining continuous flow conditions and using a low temperature. Furthermore, the Michaelis–Menten model of enzyme kinetics works under the assumption that [E] << [S], which might not reflect the conditions used to collect SPR binding data, as protein is localized on the chip surface. So, while SPR is a valuable tool for identifying potential and preferred targets of ADO, it does not provide accurate information on the specific kinetic factors which dictate protein substrate selectivity.

Experiments with full-length RGS5 indicate that the globular structure of the protein does not significantly contribute to substrate binding and catalytic efficiency relative to representative peptide, with site directed *N*t-cys mutants and modifications reducing (C2S) and eliminating (C2A and RGS5-Ox) association. Kinetically, this is driven by a lower *k*_cat_, suggesting that full-length RGS5 binding can influence ADO turnover, possibly by limiting O_2_ diffusion into the active site through increased steric bulk. NMR analysis of the interaction revealed that almost all binding events are restricted to the *N* terminus of RGS5, which adopts an extended loop structure according to an unpublished solution structure (PDB: 2CRP) and AlphaFold models. This arrangement may prevent ADO from interacting with the main body of RGS5, which correlates with our SPR and kinetic data. Nevertheless, ADO may form larger interfaces with other protein substrates that have shorter, less exposed *N* termini. Unfortunately, the most influential RGS5 residues identified through SPR and enzyme assays could not be unambiguously assigned to the NMR spectra due to peak overlaps.

Collectively these results suggest that an accessible, unmodified *N*t-cys is the most important feature when looking for ADO protein substrates, with adjacent residues and globular structure having moderate and limited impact on association and catalytic efficiency, respectively, at least in terms of RGS5. Nevertheless, other properties, including compatibility with downstream processing machinery such as the arginyltransferase ATE1, need to be considered when investigating potential Cys/Arg *N*-degron targets. The role of iron in mediating substrate binding and turnover needs to be explored in more detail when considering protein targets, with an X-ray crystal structure of an enzyme:substrate complex highly valuable to this goal. This will help elucidate enzyme mechanism and develop chemical strategies to modulate ADO activity in the context of hypoxic disease.

## Experimental procedures

### Protein production

#### Protein expression

Full-length human ADO (UniProt ID: Q96SZ5) was cloned into the pET28a (thrombin cleavage site following tag) and pETDuet (TEV cleavage site following tag) plasmids for bacterial expression as *N*-terminal His-tagged proteins and into the pQE80L-Navi plasmid for bacterial expression as an *N* terminally His-tagged and biotinylated protein. The pET28a-ADO construct was used to produce protein for enzyme kinetic assays, the pETDuet-ADO construct was used to produce protein for NMR titrations, and the pQE80L-Navi-ADO construct was used to produce protein for SPR experiments. Full-length human RGS5 (UniProt ID: O15539) was cloned into pETDuet with an *N*-terminal His- and SUMO-tag for bacterial expression. The pETDuet-His-SUMO-RGS5 construct was used to produce all RGS5 proteins used in this study. Site-directed mutagenesis was used to introduce mutations in the constructs listed above.

All constructs were transformed into Rosetta2(DE3) *Escherichia coli* (*E. coli*) cells in preparation for protein expression. 2xYT, supplemented with the appropriate antibiotics, was used as the expression medium to express standard proteins. Expression cultures were inoculated with saturated overnight cultures (1:100 dilution) prepared using single colonies from fresh transformations or glycerol stocks. Expression cultures were incubated at 37 °C, with shaking at 120 to 150 rpm, and allowed to grow to an *A*_600_ of ∼0.6 to 0.8 before being cooled to room temperature and supplemented with 0.5 mM IPTG (and 0.2 mM biotin for the pQE80L-Navi construct to enable biotinylation) to induce expression. Expression cultures were further incubated at 20 °C, with shaking, for ∼18 to 24 h before harvesting *via* centrifugation at 4000*g* for 25 min. Cell pellets were stored at −20 °C until required for protein purification.

Uniformly ^13^C/^15^N-labeled proteins and metal-exchanged proteins were produced by as described above for standard proteins up until and *A*_600_ of ∼0.6 to 0.8 was reached. At this point, cultures were harvested (15 min at 4000*g*) and cell pellets were resuspended in either minimal medium routinely used for isotopic labelling of recombinant proteins for ^13^C/^15^N-labeled proteins or M9 minimal media for metal-exchanged proteins (37). A volume of minimal media half the volume of the original 2×YT expression culture was used for resuspension and the appropriate antibiotics were added. Minimal media used for ^13^C/^15^N-labeled proteins was supplemented with ^13^C-glucose and/or ^15^N-ammonium chloride and minimal media used for making metal-exchanged proteins was supplemented with 0.2 mM Co^2+^ or Zn^2+^. The minimal media expression cultures were then transferred to 20 °C, with shaking, for 45 min before expression was induced with 0.5 mM IPTG and allowed to proceed as described above.

#### Protein purification

##### ADO prepared for enzyme assays (pET28a-ADO)

A purification procedure of nickel-ion (Ni^2+^) affinity chromatography followed by size-exclusion chromatography was used to prepare the ADO used for enzyme assays in this study. Cell pellets were resuspended in lysis buffer (50 mM Tris pH 7.5, 400 mM NaCl, 20 mM imidazole, 10 μg/ml DNAse I, and 1× cOmplete EDTA-free protease inhibitor) and lysed by sonication before being clarified *via* centrifugation (17,000*g* for 30 min followed by filtration through a 0.45 μM membrane). The soluble fractions of cell lysates were subject to Ni^2+^-affinity chromatography by passing the supernatant through a HisTrap HP column (Cytiva) equilibrated in wash buffer (50 mM Tris pH 7.5, 400 mM NaCl, 20 mM imidazole). Bound proteins were washed and eluted using a 20 mM to 1 M imidazole gradient in a base buffer comprised of 50 mM Tris pH 7.5 and 400 mM NaCl. Protein containing fractions were concentrated and buffer exchanged into size exclusion chromatography (SEC) buffer (50 mM Tris pH 7.5 and 400 mM NaCl) using a PD-10 desalting column (Cytiva). The desalted protein was injected onto equilibrated 26/600 HiLoad Superdex 75 prep grade column (Cytiva) and eluted using SEC buffer. Protein purity was assessed by SDS-PAGE and concentrations were determined using *A*_280_ nm measurements.

##### ADO prepared for SPR experiments

A purification procedure of nickel-ion (Ni^2+^) affinity chromatography followed by size-exclusion chromatography was used to prepare the ADO used SPR in this study. Cell pellets were resuspended in lysis buffer (20 mM Hepes pH 7.5, 500 mM NaCl, 1 mM tris(2-carboxyethyl)phosphine (TCEP), 20 mM imidazole, 10 μg/ml DNAse I, 100 μg/ml lysozyme, and 1× cOmplete EDTA-free protease inhibitor) and lysed by sonication before being clarified *via* centrifugation (18,000*g* for 30). The soluble fractions of cell lysates were subject to Ni^2+^-affinity chromatography by incubating the supernatant with Ni-NTA agarose (Cytiva) equilibrated in wash buffer (20 mM Hepes pH 7.5, 500 mM NaCl, 1 mM TCEP, and 20 mM imidazole). Bound proteins were washed and eluted with elution buffer (20 mM Hepes pH 7.5, 500 mM NaCl, 1 mM TCEP, and 500 mM imidazole). Protein containing fractions were concentrated injected onto an equilibrated 26/600 HiLoad Superdex 75 prep grade column (Cytiva) and eluted using SEC buffer (20 mM Hepes pH 7.5, 150 mM NaCl, and 1 mM TCEP). Protein purity was assessed by SDS-PAGE and concentrations were determined using *A*_280_ nm measurements.

Co^2+^-affinity chromatography, using Talon resin (Cytiva), was used instead of Ni^2+^ to purify metal-exchanged ADO proteins used for SPR with the same procedure described above used but with different lysis (20 mM Hepes pH 7.5, 500 mM NaCl, 0.1 μM CoCl_2_/ZnSO_4_, 2.5 mM imidazole, 10 μg/ml DNAse I, 100 μg/ml lysozyme, and 1× cOmplete EDTA-free protease inhibitor), wash (20 mM Hepes pH 7.5, 500 mM NaCl, 0.1 μM CoCl_2_/ZnSO_4_, and 2.5 mM imidazole), and elution buffers (20 mM Hepes pH 7.5, 500 mM NaCl, 1 mM TCEP, and 300 mM imidazole).

##### ADO prepared for NMR experiments

A purification procedure of nickel-ion (Ni^2+^) affinity chromatography, followed by TEV cleavage for His-tag removal, and size-exclusion chromatography was used to prepare the ADO used NMR in this study. Cell pellets were resuspended in lysis buffer (20 mM Hepes pH 7.5, 500 mM NaCl, 1 mM TCEP, 20 mM imidazole, 10 μg/ml DNAse I, 100 μg/ml lysozyme, and 1× cOmplete EDTA-free protease inhibitor) and lysed by sonication before being clarified *via* centrifugation (18,000*g* for 30). The soluble fractions of cell lysates were subject to Ni^2+^-affinity chromatography by incubating the supernatant with Ni-NTA agarose (Cytiva) equilibrated in wash buffer (20 mM Hepes pH 7.5, 500 mM NaCl, 1 mM TCEP, and 20 mM imidazole). Bound proteins were washed and eluted with elution buffer (20 mM Hepes pH 7.5, 500 mM NaCl, 1 mM TCEP, and 500 mM imidazole). Eluates were concurrently dialysed in SEC buffer (20 mM Hepes pH 7.5, 150 mM NaCl, and 1 mM TCEP) and incubated with TEV protease overnight at 4 °C to cleave the His-tag. The dialyzed cleaved protein was passed through Talon resin (Cytiva) for His-tag removal before being concentrated and injected onto an equilibrated 16/600 HiLoad Superdex 75 SEC column (Cytiva). Protein was eluted from the column using SEC buffer. Protein purity was assessed by SDS-PAGE and concentrations were determined using *A*_280_ nm measurements.

Co^2+^-affinity chromatography, using Talon resin (Cytiva), was used instead of Ni^2+^ to purify metal-exchanged ADO proteins used for NMR with the same procedure described above used but with different lysis (20 mM Hepes pH 7.5, 500 mM NaCl, 0.1 μM CoCl_2_/ZnSO_4_, 2.5 mM imidazole, 10 μg/ml DNAse I, 100 μg/ml lysozyme, and 1× cOmplete EDTA-free protease inhibitor), wash (20 mM Hepes pH 7.5, 500 mM NaCl, 0.1 μM CoCl_2_/ZnSO_4_, and 2.5 mM imidazole), and elution buffers (20 mM Hepes pH 7.5, 500 mM NaCl, 1 mM TCEP, and 300 mM imidazole).

##### RGS5 purification

The same protocol was used to purify RGS5 for all experiments described in this paper. A purification procedure consisting of nickel-ion (Ni^2+^) affinity chromatography, followed by Ulp1 cleavage for His-SUMO-tag removal, and size-exclusion chromatography was used to prepare RGS5. Cell pellets were resuspended in lysis buffer (20 mM Hepes pH 7.5, 500 mM NaCl, 1 mM TCEP, 20 mM imidazole, 10 μg/ml DNAse I, 100 μg/ml lysozyme, and 1× cOmplete EDTA-free protease inhibitor) and lysed by sonication before being clarified *via* centrifugation (18,000*g* for 30). The soluble fractions of cell lysates were subject to Ni^2+^-affinity chromatography by incubating the supernatant with Ni-NTA agarose (Cytiva) equilibrated in wash buffer (20 mM Hepes pH 7.5, 500 mM NaCl, 1 mM TCEP, and 20 mM imidazole). Bound proteins were washed and eluted with elution buffer (20 mM Hepes pH 7.5, 500 mM NaCl, 1 mM TCEP, and 500 mM imidazole). Eluates were concurrently dialyzed in SEC buffer (20 mM Hepes pH 7.5, 150 mM NaCl, and 1 mM TCEP) and incubated with Ulp1 protease overnight at 4 °C to cleave the His-SUMO-tag. The dialyzed cleaved protein was passed through Talon resin (Cytiva) for His-SUMO-tag removal before being concentrated and injected onto an equilibrated 16/600 HiLoad Superdex 75 SEC column (Cytiva). Protein was eluted from the column using SEC buffer. Protein purity was assessed by SDS-PAGE and concentrations were determined using *A*_280_ nm measurements.

### Peptide synthesis

#### General materials and methods

Peptide grade *N,N*-dimethylformamide (DMF) for peptide synthesis was purchased from RCI. SupraGradient grade acetonitrile (MeCN) for chromatography was purchased from Scharlau and pure water (type 1) was obtained from a Merck Millipore Direct-Q 5 water purification system. Fmoc-protected amino acids (Fmoc-Xaa-OH), coupling reagents, and resins were purchased from Mimotopes. SPPS was performed using automated synthesis on a Biotage Syro I peptide synthesizer. All other reagents were purchased from Sigma-Aldrich and used as received.

#### Fmoc-solid-phase peptide synthesis

##### Automated peptide synthesis (SYRO I peptide synthesizer)

The resin (90 mg, 50 μmol, 0.57 mmol g^−1^) was treated with 40 vol.% piperidine in DMF (800 μl) for 4 min, drained, then treated with 20 vol.% piperidine in DMF (800 μl) for 4 min, drained, and washed with DMF (4 × 2.2 ml). The resin was then treated with a solution of Fmoc-Xaa-OH and Oxyma (220 μmol, 4.4 eq.) in DMF (400 μl), a 1 wt.% solution of 1,3-di*iso*propyl-2-thiourea in DMF (400 μl), followed by a solution of N,N′-Diisopropylcarbodiimide (200 μmol, 4 eq.) in DMF (400 μl). All couplings were performed twice and carried out at 40 °C for 45 min. The resin was then drained and washed with DMF (1 × 1.2 ml) before being treated with a solution of 2.5 vol.% Ac_2_O and 5 vol.% *i*Pr_2_NEt in DMF (800 μl) for 6 min at room temperature, drained, and washed with DMF (4 × 1.25 ml).

##### Manual cleavage

The resin was thoroughly washed with CH_2_Cl_2_ (5 × 5 ml) before being treated with 90:5:5 v/v/v TFA:tri*iso*propylsilane:H_2_O and shaken at room temperature for 2 h. The resin was filtered and the filtrate concentrated under a stream of nitrogen before addition of diethyl ether (35 ml). The peptide was pelleted by centrifugation (4 min, 2 °C, at 5800*g*) and the diethyl ether was decanted. The crude peptide was dissolved in dimethylsulfoxide (1.2 ml) in preparation for HPLC purification.

##### HPLC purification

All HPLC purification was conducted on a Shimadzu Mass-Directed HPLC with an LCMS-2020 (ESI) single quad mass spectrometer. All peptides were purified on a Waters XBridge Peptide BEH C18 OBD prep column (130 Å, 5 um, 19 mm × 150 mm) at a flow rate of 15 ml/min using a mobile phase of 0.1% formic acid in water (solvent A) and 0.1% formic acid in MeCN (solvent B). All peptides were purified with a linear gradient from 1 to 60% solvent B over 30 min. Clean fractions were concentrated by lyophilization to give a white solid.

### Surface plasmon resonance

SPR measurements were made using a Biacore T200 (Cytiva) instrument and data were analyzed using the Biacore Insight Evaluation Software (Cytiva; https://www.cytivalifesciences.com/en/us/shop/protein-analysis/spr-label-free-analysis/spr-software-and-extensions/biacore-insight-evaluation-software-p-23528). Biotinylated-ADO was immobilized on a Biotin CAP chip (Cytiva) with a target density of ∼2500 to 3000 RU and peptide/protein substrates were injected over the chip. Experiments were conducted at 4 °C using both multicycle (fit using the equilibrium steady state affinity 1:1 binding model) and single cycle kinetics mode (fit using a 1:1 binding model). A buffer comprising 20 mM Hepes pH 7.5, 500 mM NaCl, 10 mM DTT, and 0.1% (v/v) tween-20 was used as the running buffer at a flow rate of 50 μl/min.

For metal-chelation experiments, a mixture of 10 mM 1, 10-phenanthroline and 100 mM EDTA (pH 8) was injected onto the chip using 10 × 60 s injections at a 100 μl/min flow rate. For iron reconstitution, a solution of 0.1 mM FeSO_4_ and 12.5 mM sodium ascorbate was injected onto the chip for 60 to 300 s at a flow rate of 2 μl/min. The instrument and chip were primed into buffer excluding reducing agents before conducting metal chelation and iron reconstitution.

### Activity assay

The enzymatic activity of ADO was examined by incubating between 0 to 1000 μM peptide and protein substrates with 0.05 to 0.1 μM ADO in a bench-top thermomixer at 37 °C for 45 s. Otherwise stated, the reaction buffer contained 50 mM Tris pH 7.5, 50 mM NaCl, and 5 mM TCEP. The reactions were quenched by mixing the sample 1:10 (peptide) or 1:5 (protein) with 1% formic acid (v/v).

Peptide and protein samples were injected onto a Chromolith RP-18 Endcapped HPLC Columns (100–2 mm; Merck) and Discovery BIO Wide Pore C5 HPLC Column (Supelco), respectively, heated to 40 °C and eluted at 0.3 ml/min using a gradient of 95% deionized water supplemented with 0.1% (v/v) formic acid to 95% acetonitrile. Oxidation was monitored by an ultra high-performance liquid chromatography MS using ExionLC AD (Sciex) and SelexION (Sciex) mass spectrometer operated in a positive electrospray TOF (+ESI-TOF) mode. Instrument parameters, data acquisition and data processing were controlled by Analyst software (Sciex; https://sciex.com/products/software/analyst-software). The peptide data processing was performed using Skyline software (MacCoss Lab Software; https://skyline.ms/project/home/software/Skyline/begin.view) (38). All figures and parameters were generated using GraphPad Prism 9 (GraphPad; https://www.graphpad.com/features).

### Inductively coupled plasma mass spectrometry

Protein samples (50 μl) were diluted in 100 μl concentrated nitric acid (trace metal free) and incubated overnight before being submitted to the School of Chemistry MS Facility for ICP-MS analysis.

### NMR spectroscopy

A sample of uniformly ^13^C- and ^15^N-labeled RGS5^FL^-C2S was prepared at 1 mM, in a buffer comprising 20 mM Hepes pH 7.5, 50 mM NaCl, 10 mM DTT, and 10% (v/v) D_2_O, for enable chemical shift assignment of the backbone atoms of the protein. NMR experiments (2D ^15^N-HSQC and 3D CBCA(CO)NH, HNCACB, and HNCA) were acquired at 25 °C using a Bruker Avance III 800 MHz NMR spectrometer fitted with a TCI probe head using standard pulse sequences from the Bruker library. NMR spectra were processed by Topspin3.2 (Bruker BioSpin) or using NMRPipe on NMRBox (https://nmrbox.nmrhub.org/). Spectra were internally referenced with respect to 10 μM 4,4-dimethyl-4-silapentane-1-sulfonic acid trimethyl signal at 0 ppm. Topspin, NMRFAM-SPARKY, CARA, and CcpNmr were used for spectral analysis. H^N^, N, C^α^, and C^β^ chemical shift assignments for RGS5^FL^-C2S were carried out by CcpNmr (39, 40, 41).

For titrations with ADO, ^15^N-labeled RGS5^FL^ proteins were prepared at 50 μM, in buffer containing 20 mM Hepes pH 7.5, 150 mM NaCl, 10 mM DTT, and 10% (v/v) D_2_O. ^15^N-HSQC spectra were collected before and after the addition of ADO to the labeled RGS5^FL^ samples. Intensity-based CSP plots were produced by calculating peak height values from the ^15^N-HSQCs before and after addition of ADO and normalizing against the heights of ∼5 peaks from the HSQC that remained invariant throughout the titration.

The random coil deviation for C^α^ and C^β^ shifts of RGS5^FL^-C2S were calculated using the following equation (40).

$$\Delta \delta_{i}=1/3\left(\Delta \delta C_{i-1}^{\alpha }+\Delta \delta C_{i}^{\alpha }+\Delta \delta C_{i+1}^{\alpha }-\Delta \delta C_{i-1}^{\beta }-\Delta \delta C_{i}^{\beta }-\Delta \delta C_{i+1}^{\beta }\right)$$

## Data availability

The chemical shift assignments for RGS5^FL^-C2S have been deposited to the Biomagnetic Resonance Bank (BMRB entry: 52267).

## Supporting information

This article contains supporting information (12, 42).

## Conflict of interest

The authors declare that they have no conflicts of interest with the contents of this article.
