# Supplementary material for: The enzymatic oxygen sensor cysteamine dioxygenase binds its protein substrates through their N-termini
Source: J Biol Chem. 2024 Aug 8;300(9):107653. doi: 10.1016/j.jbc.2024.107653 (PMC11406360; doi:10.1016/j.jbc.2024.107653)
Supplement: Supplemental Tables S1–S8 and Figures S1–S15 [file mmc1.docx]

**SUPPORTING INFORMATION**

The oxygen-sensing enzyme cysteamine dioxygenase binds its protein substrates through their *N*-termini

Karishma Patel^1, 2^, Yannasittha Jiramongkol^1, 3^, Alexander Norman^1^, Joshua W.C. Maxwell^1^, Biswaranjan Mohanty^4^, Richard J. Payne^1^, Kristina M. Cook^5^, Mark D. White^1*^

^1^School of Chemistry, The University of Sydney, NSW 2006 Australia

^2^School of Life and Environmental Sciences, The University of Sydney, NSW 2006 Australia

^3^Faculty of Science, Charles Perkins Centre, The University of Sydney, NSW 2006, Australia

^4^Sydney Analytical Core Research Facility, The University of Sydney, NSW 2006, Australia

^5^Faculty of Medicine and Health, Charles Perkins Centre, The University of Sydney, NSW 2006, Australia

*To whom correspondence should be addressed:

MDW: mark.white@sydney.edu.au

**LIST OF MATERIALS INCLUDED**

**INCLUDED IN THIS DOCUMENT:**

- **Supplementary Tables S1 – S8**
- **Supplementary Figure S1 – S15**

**TABLES**

**Table S1.** Equilibrium dissociation constants for the binding of modified *N*t-cys RGS5 peptides to ADO.


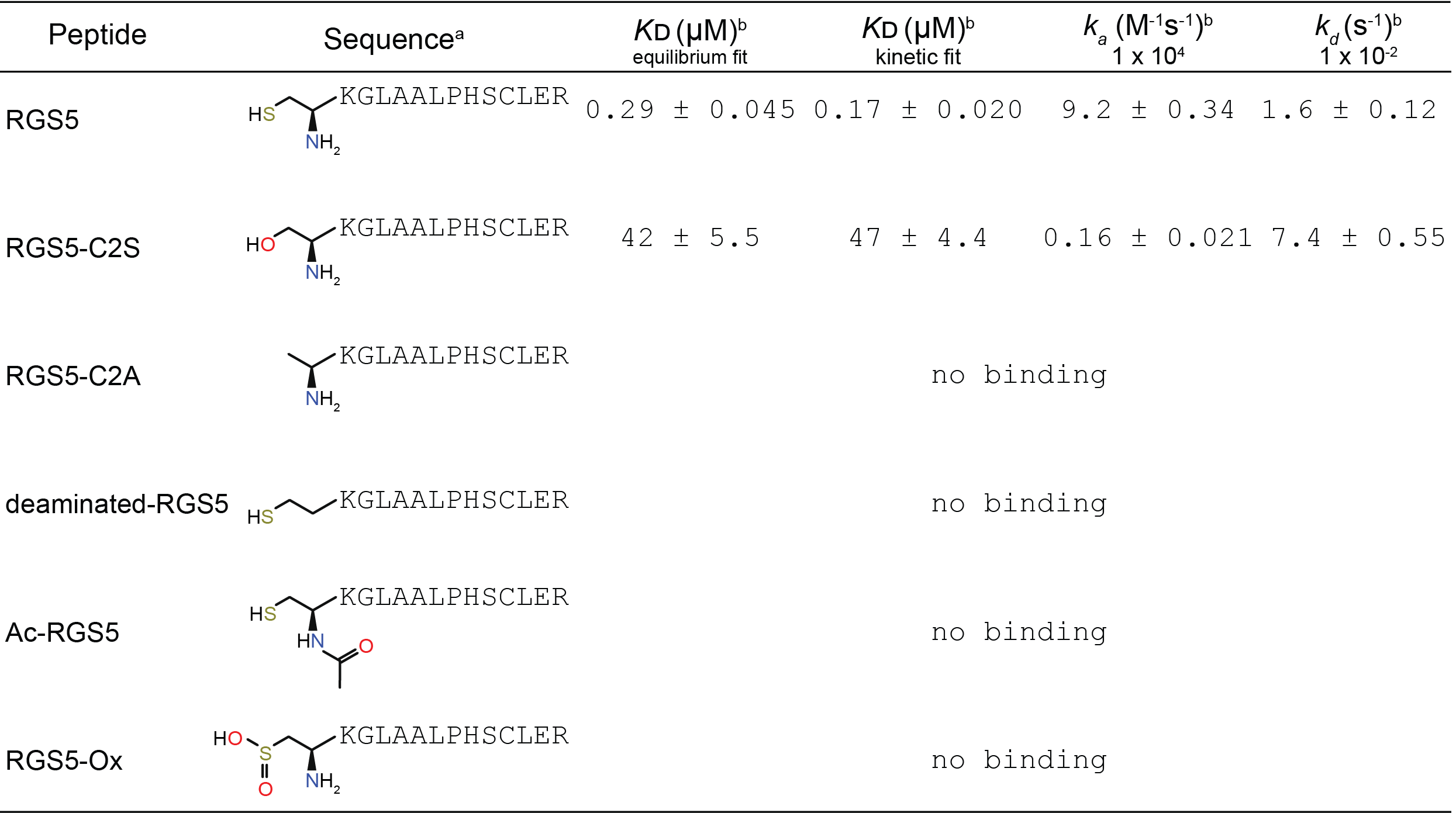


^a^ The chemical structures of the *N*t-cys modifications are displayed

^b^ The values are given as the geometric mean (± standard deviation) of a minimum of three independent SPR measurements.

**Table S2.** Equilibrium dissociation constants for the binding of modified *N*t-cys IL32 peptides to ADO.


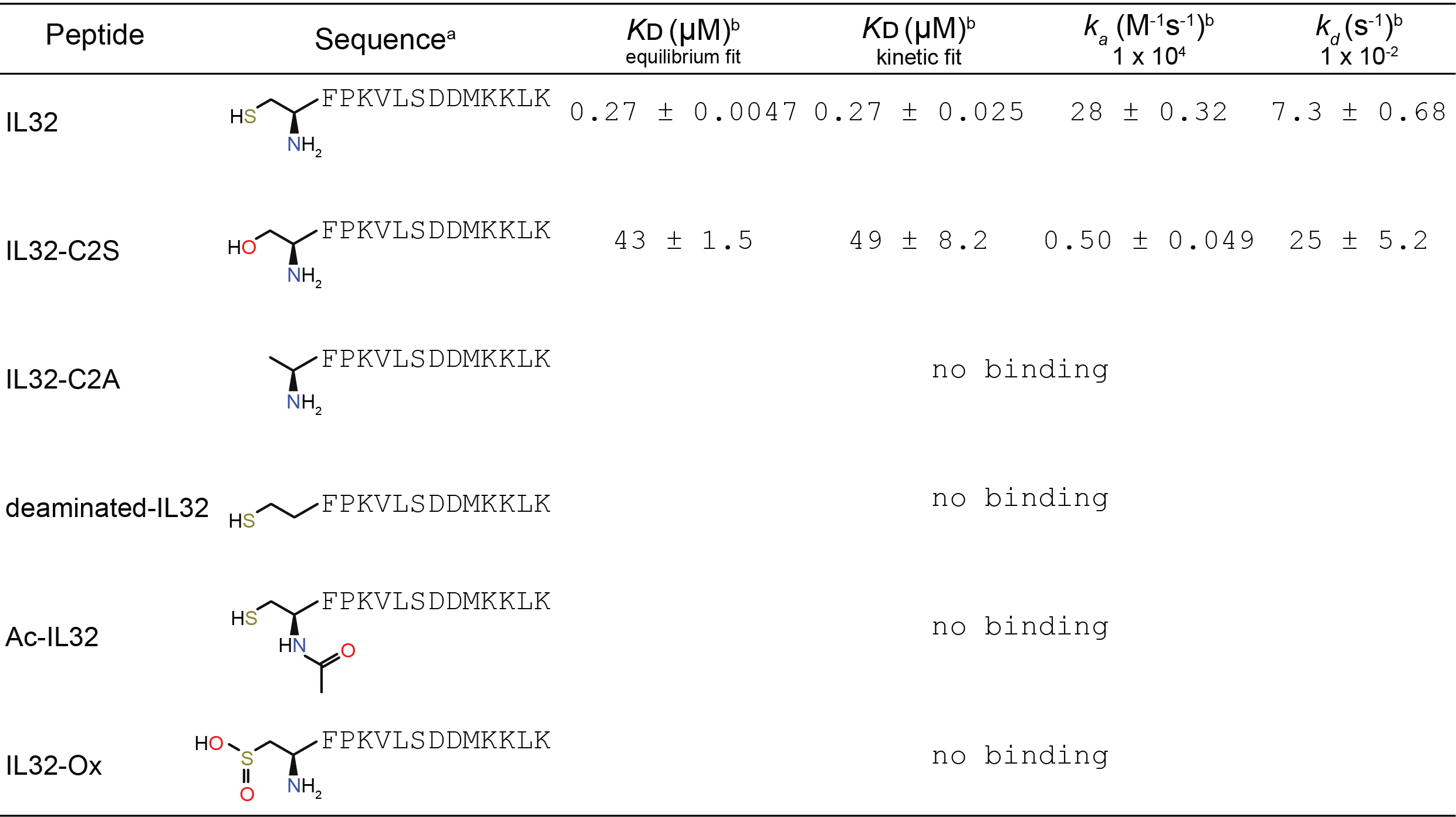


^a^ The chemical structures of the *N*t-cys modifications are displayed.

^b^ The values are given as the geometric mean (± standard deviation) of a minimum of three independent SPR measurements.

**Table S3**. Equilibrium dissociation constants for the binding of RGS5 and IL32 peptides to metal-deficient and metal-exchanged ADO proteins.


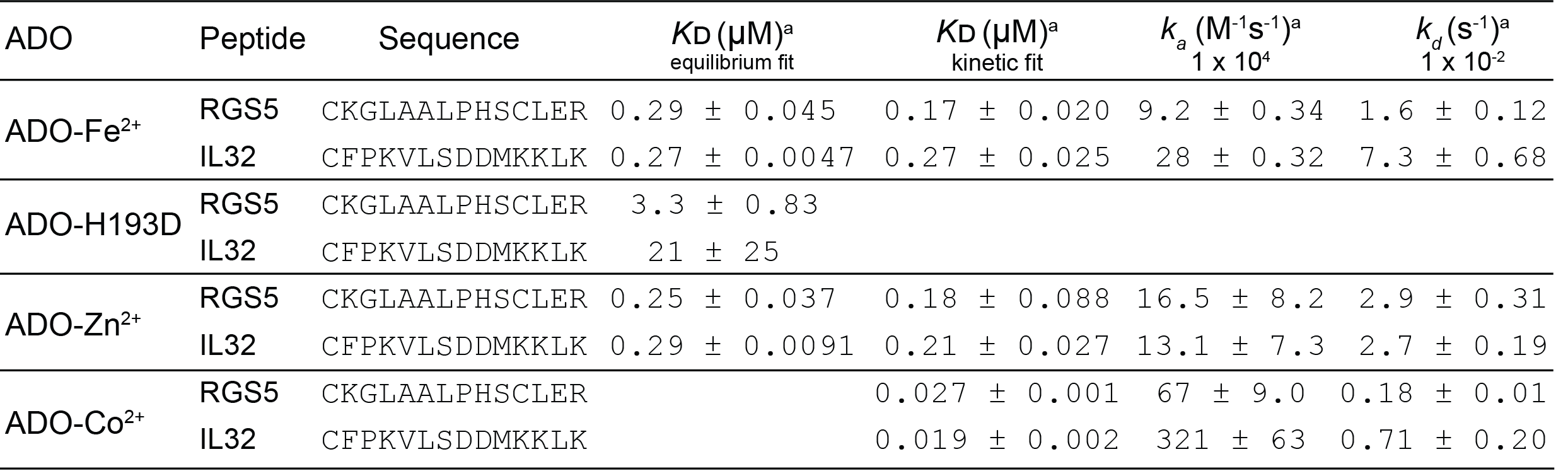


^a^ The values are given as the geometric mean (± standard deviation) of a minimum of three independent SPR measurements. The values of iron-incorporated wildtype ADO with RGS5 and IL32 are provided for comparison.

**Table S4.** Equilibrium dissociation constants for the binding of the RGS5 alanine scan peptides with ADO.


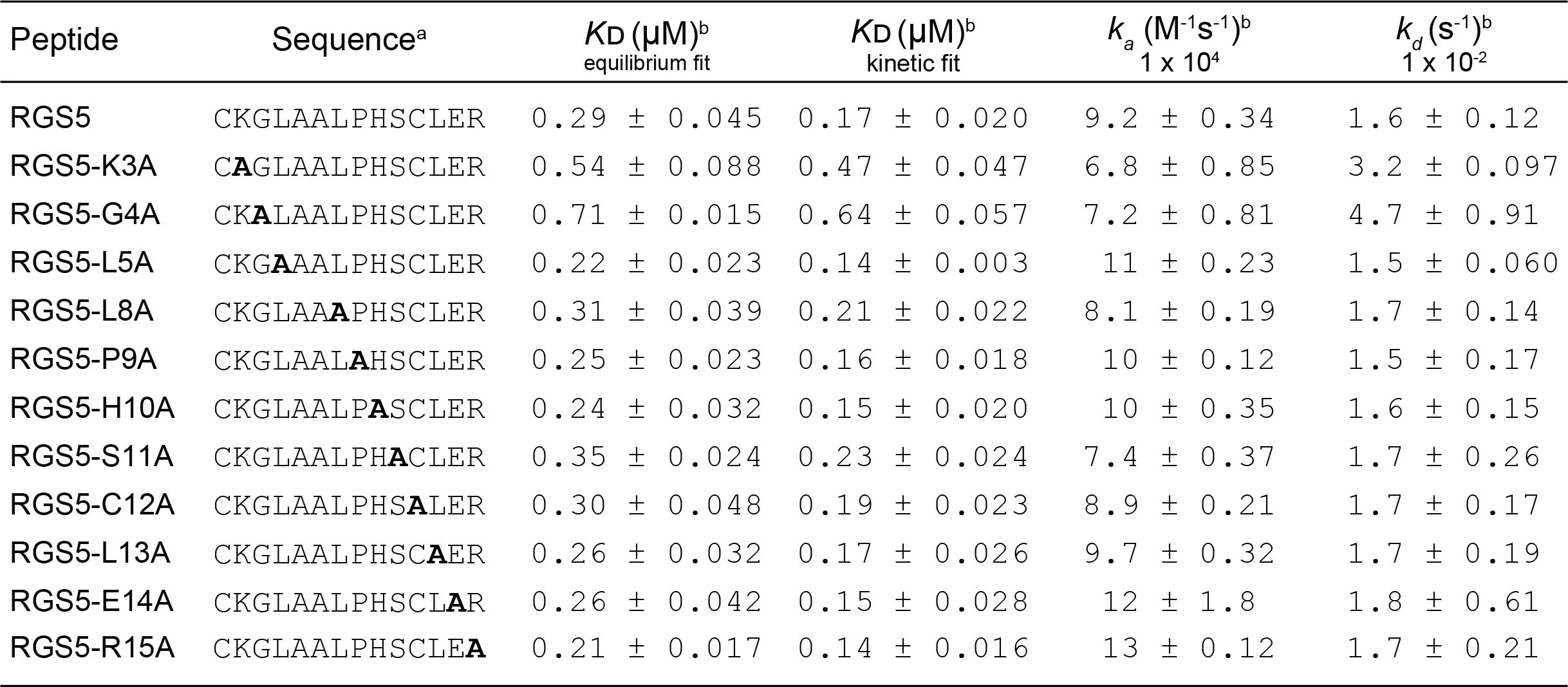


^a^ The alanine mutation in each peptide is shown in **bold**.

^b^ The values are given as the geometric mean (± standard deviation) of a minimum of three independent SPR measurements. The values for unmodified RGS5 are provided for comparison.

**Table S5.** Equilibrium dissociation constants for the binding of the IL32 alanine scan peptides with ADO.


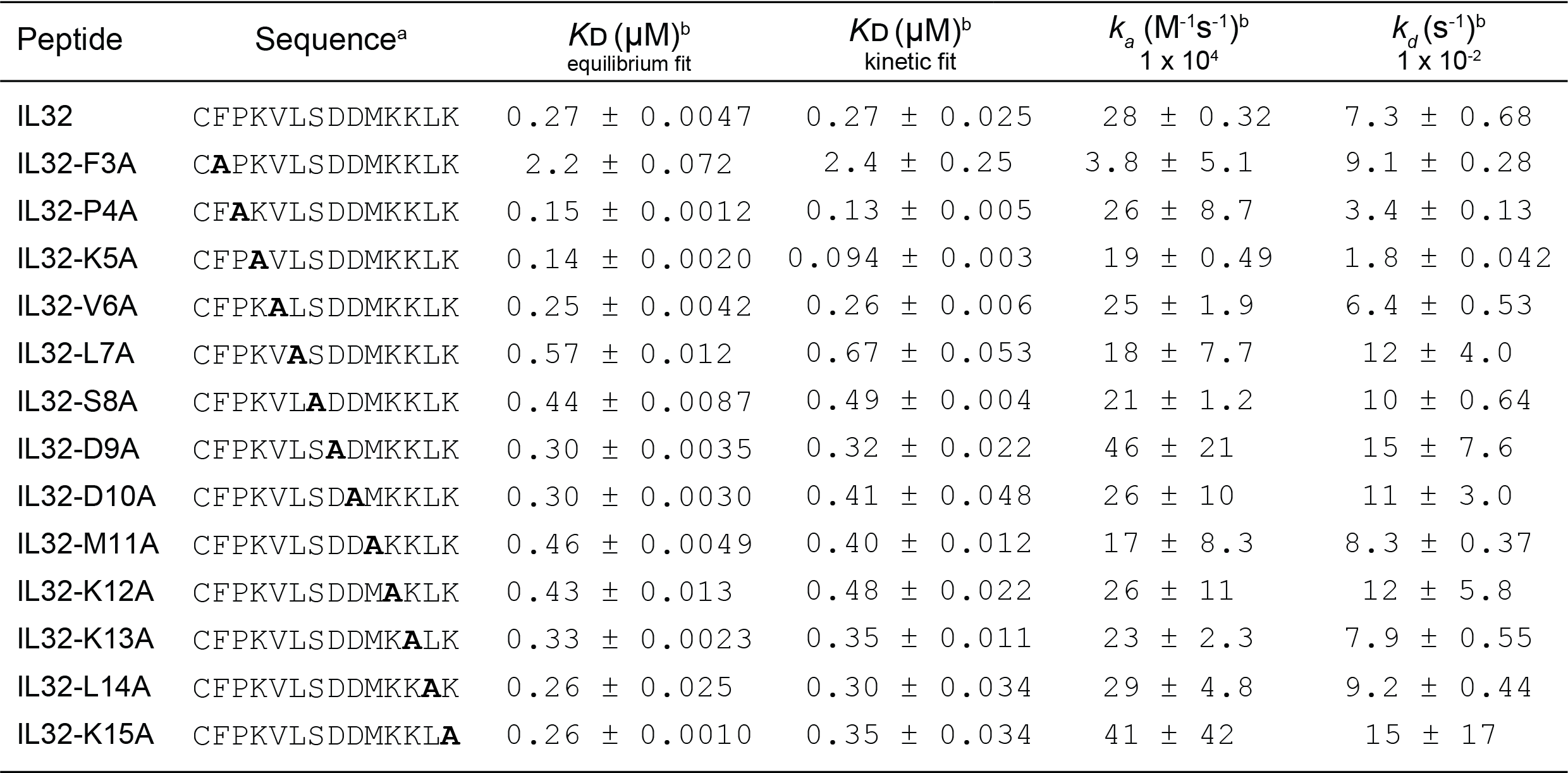


^a^ The alanine mutation in each peptide is shown in **bold**.

^b^ The values are given as the geometric mean (± standard deviation) of a minimum of three independent SPR measurements. The values for unmodified IL32 are provided for comparison.

**Table S6**. Equilibrium dissociation constants for the binding of short RGS5 and

IL32 peptides with ADO.


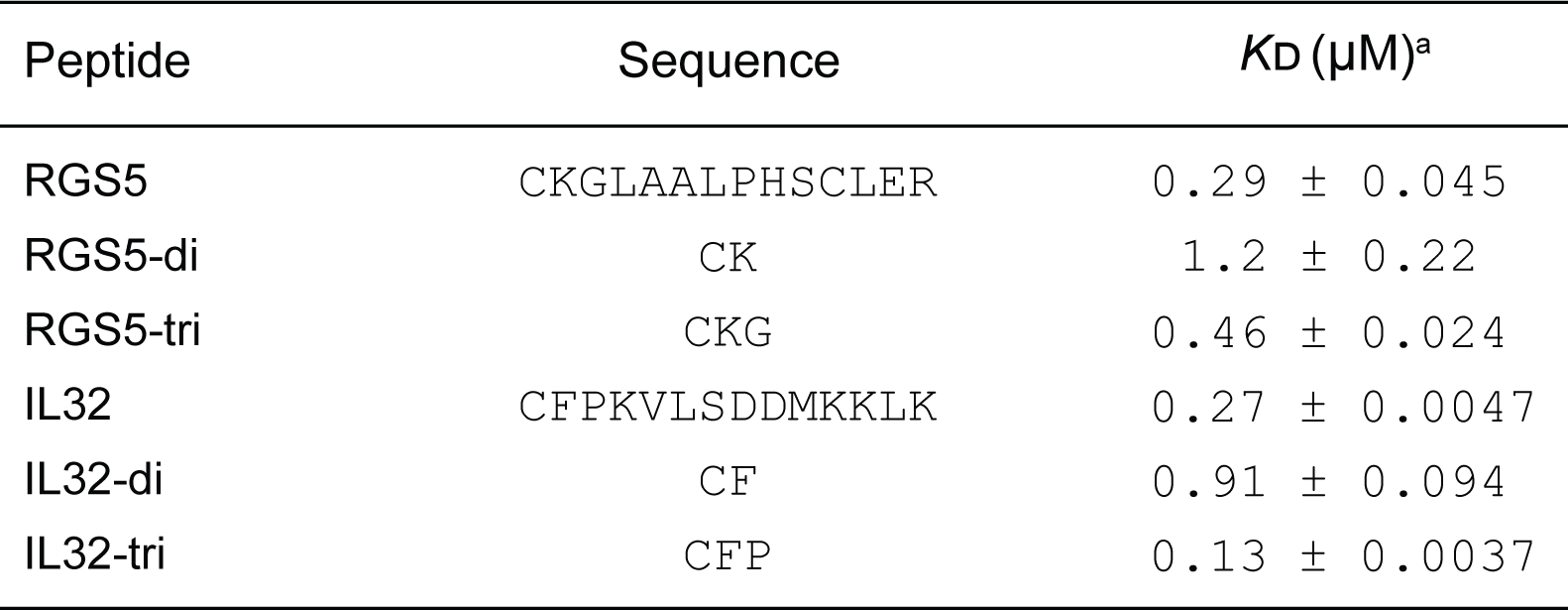


^a^ The *K*ᴅ values are given as the geometric mean (± standard deviation) of a

minimum of three independent SPR measurements. The *K*ᴅ values of iron-

incorporated wildtype ADO with RGS5 and IL32 is provided for comparison.

**Table S7.** Equilibrium dissociation constants for the binding of the RGS5 third and fourth position mutant peptides with ADO.

**
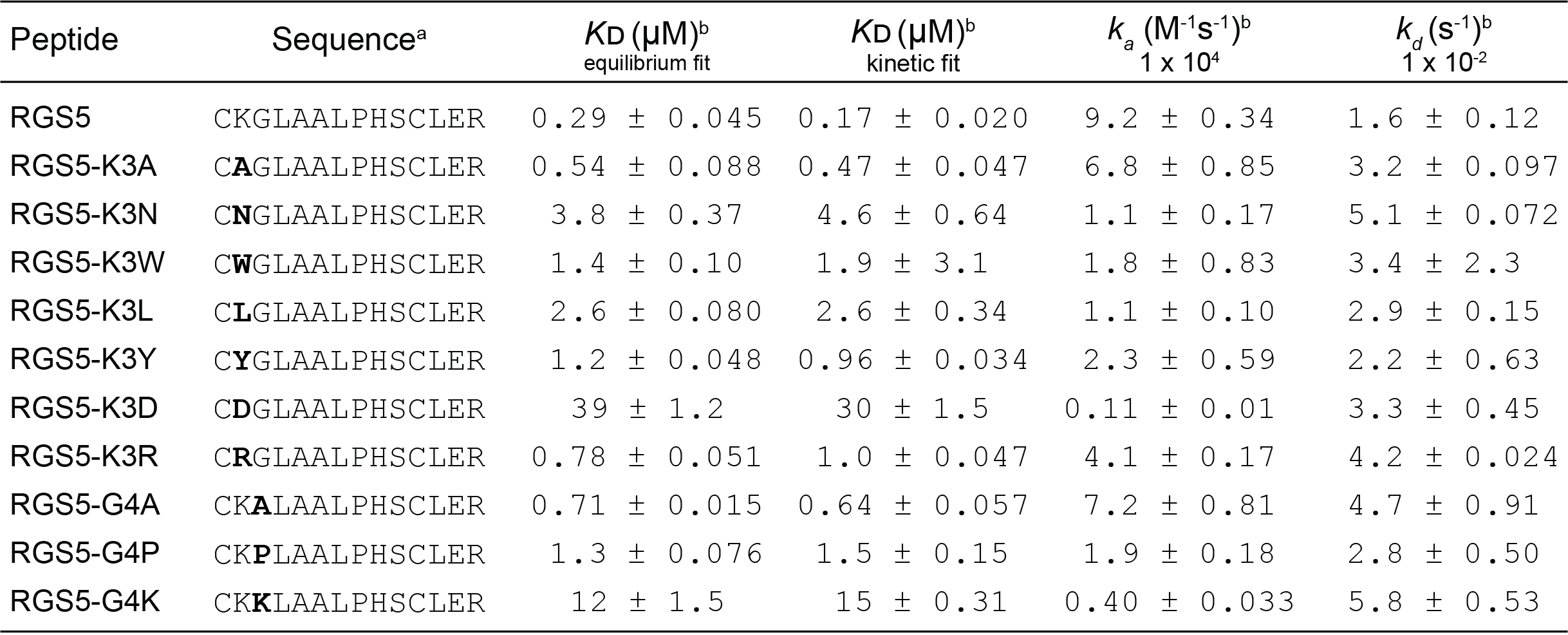
**

^a^ The mutations in each peptide is shown in **bold**.

^b^ The values are given as the geometric mean (± standard deviation) of a minimum of three independent SPR measurements.

**Table S8**. Random coil deviation for C^α^ and C^β^ shifts of RGS5^FL^-C2S.

| Amino acid | | C^α^ [ppm] | C^β^ [ppm] | (C^α^ - C^β^)[ppm] | Secondary Structure |
| --- | --- | --- | --- | --- | --- |
| MET | 1 | 0 | 0 | 0 |  |
| SER | 2 | 0 | 0 | 0 |  |
| LYS+ | 3 | -0.576 | 0.032 | -0.28 |  |
| GLY | 4 | -0.233 | 0 | -0.507 |  |
| LEU | 5 | -0.663 | 0.016 | -0.664 |  |
| ALA | 6 | -0.933 | 0.148 | -1.117 | STRAND |
| ALA | 7 | -1.324 | 0.268 | -0.859 |  |
| LEU | 8 | -0.701 | -0.798 | -0.498 |  |
| PRO | 9 | 0 | 0 | 0 |  |
| HIS | 10 | 0 | 0 | 0 |  |
| SER | 11 | 0 | 0 | 0 |  |
| CYS | 12 | 0 | 0 | 0 |  |
| LEU | 13 | 0.986 | -0.506 | 0.839 |  |
| GLU- | 14 | 0.462 | -0.563 | 1.189 | HELIX |
| ARG+ | 15 | 0.554 | -0.495 | 0.996 | HELIX |
| ALA | 16 | 0.464 | -0.449 | 0.918 |  |
| LYS+ | 17 | 0.704 | -0.088 | 0.641 |  |
| GLU- | 18 | 0.127 | -0.09 | 0.635 |  |
| ILE | 19 | 0.6 | -0.296 | 0.456 |  |
| LYS+ | 20 | 0.234 | -0.021 | 0.343 |  |
| ILE | 21 | -0.895 | -0.772 | 0.151 |  |
| LYS+ | 22 | 0.054 | -0.266 | 0.012 |  |
| LEU | 23 | -0.246 | -0.086 | 0.154 |  |
| GLY | 24 | 0.302 | 0 | 0.037 |  |
| ILE | 25 | -0.076 | -0.045 | -0.077 |  |
| LEU | 26 | -0.507 | -0.005 | -0.321 |  |
| LEU | 27 | -0.546 | -0.115 | 0.472 |  |
| GLN | 28 | 5.597 | 3.248 | -0.197 |  |
| LYS+ | 29 | 3.186 | 5.694 | -0.051 |  |
| PRO | 30 | -0.008 | -0.014 | -0.971 |  |
| ASP- | 31 | -0.37 | 0.042 | -0.346 |  |
| SER | 32 | -0.625 | 0.007 | -0.256 |  |
| VAL | 33 | 0.006 | -0.271 | -0.173 |  |
| GLY | 34 | -0.165 | 0 | -0.196 |  |
| ASP- | 35 | -0.439 | 0.261 | -0.507 |  |
| LEU | 36 | -0.643 | 0.013 | -0.641 |  |
| VAL | 37 | -0.483 | 0.085 | -1.492 | STRAND |
| ILE | 38 | -1.66 | 1.593 | -1.353 | STRAND |
| PRO | 39 | -0.252 | -0.014 | -0.924 |  |
| TYR | 40 | -0.042 | -0.761 | -0.212 |  |
| ASN | 41 | -0.803 | 0.313 | -0.539 |  |
| GLU- | 42 | -0.954 | 0.267 | -0.976 | STRAND |
| LYS+ | 43 | -1.007 | -0.415 | -0.716 |  |
| PRO | 44 | -0.349 | -0.014 | -0.739 |  |
| GLU- | 45 | -0.99 | 0.299 | -0.768 |  |
| LYS+ | 46 | -1.057 | -0.377 | -0.817 |  |
| PRO | 47 | -0.398 | 0.084 | -0.761 |  |
| ALA | 48 | -0.892 | 0.229 | -0.833 |  |
| LYS+ | 49 | -0.716 | 0.181 | -0.807 |  |
| THR | 50 | -0.216 | 0.188 | -0.899 |  |
| GLN | 51 | -1.14 | 0.256 | -0.89 |  |
| LYS+ | 52 | -0.549 | 0.32 | -0.951 | STRAND |
| THR | 53 | -0.31 | 0.279 | -0.856 |  |
| SER | 54 | -0.728 | 0.382 | 0.174 |  |
| LEU | 55 | 1.612 | -0.61 | 1.165 | HELIX |
| ASP- | 56 | 1.981 | -0.402 | 2.116 | HELIX |
| GLU- | 57 | 1.133 | -0.611 | 1.845 | HELIX |
| ALA | 58 | 0.169 | -1.238 | 1.826 | HELIX |
| LEU | 59 | 1.724 | -0.602 | 1.697 | HELIX |
| GLN | 60 | 0.781 | -0.577 | 1.047 | HELIX |
| TRP | 61 | -1.391 | -0.847 | 0.928 |  |
| ARG+ | 62 | 1.319 | -0.65 | -0.516 |  |
| ASP- | 63 | -1.35 | 1.623 | -1.057 | STRAND |
| SER | 64 | -2.52 | -0.353 | -1.263 | STRAND |
| LEU | 65 | 1.35 | 0 | 0.927 |  |
| ASP- | 66 | 3.139 | -0.458 | 2.328 | HELIX |
| LYS+ | 67 | 1.882 | -0.154 | 3.09 | HELIX |
| LEU | 68 | 2.73 | -0.907 | 2.715 | HELIX |
| LEU | 69 | 0.527 | -1.946 | 1.803 | HELIX |
| GLN | 70 | -1.045 | -0.345 | -0.978 | STRAND |
| ASN | 71 | -1.112 | 3.594 | -1.802 | STRAND |
| ASN | 72 | 0 | 0 | 0 |  |
| TYR | 73 | 2.478 | -2.03 | 2.324 | HELIX |
| GLY | 74 | 2.465 | 0 | 3.744 | HELIX |
| LEU | 75 | 3.397 | -0.862 | 3.234 | HELIX |
| ALA | 76 | 2.067 | -0.912 | 3.843 | HELIX |
| SER | 77 | 3.457 | -0.834 | 3.019 | HELIX |
| PHE | 78 | 1.425 | -0.362 | 3.511 | HELIX |
| LYS+ | 79 | 3.742 | -0.712 | 3.54 | HELIX |
| SER | 80 | 3.192 | -1.188 | 4.081 | HELIX |
| PHE | 81 | 2.27 | -1.14 | 3.904 | HELIX |
| LEU | 82 | 1.809 | -2.112 | 3.611 | HELIX |
| LYS+ | 83 | 2.501 | -1.002 | 3.497 | HELIX |
| SER | 84 | 2.153 | -0.913 | 1.664 | HELIX |
| GLU- | 85 | -2.134 | -0.556 | 2.113 | HELIX |
| PHE | 86 | 0.564 | -4.287 | 0.242 |  |
| SER | 87 | -1.759 | 0.787 | 1.555 | HELIX |
| GLU- | 88 | 1.509 | -0.852 | 1.734 | HELIX |
| GLU- | 89 | 2.992 | -2.396 | 3.58 | HELIX |
| ASN | 90 | 2.837 | -0.153 | 3.072 | HELIX |
| LEU | 91 | 0.827 | -0.011 | 2.467 | HELIX |
| GLU- | 92 | 2.611 | -0.963 | 2.385 | HELIX |
| PHE | 93 | 1.633 | -1.111 | 4.475 | HELIX |
| TRP | 94 | 7.043 | -0.064 | 4.461 | HELIX |
| ILE | 95 | 3.169 | -0.364 | 4.675 | HELIX |
| ALA | 96 | 1.862 | -1.523 | 4.177 | HELIX |
| CYS | 97 | 5.614 | 0 | 3.759 | HELIX |
| GLU- | 98 | 1.153 | -1.125 | 3.731 | HELIX |
| ASP- | 99 | 2.426 | -0.876 | 2.988 | HELIX |
| TYR | 100 | 2.498 | -0.886 | 2.863 | HELIX |
| LYS+ | 101 | 1.364 | -0.539 | 1.345 | HELIX |
| LYS+ | 102 | -1.12 | 0.131 | 1.095 | HELIX |
| ILE | 103 | 1.927 | -0.705 | -0.045 |  |
| LYS+ | 104 | -0.932 | 0.584 | -0.582 |  |
| SER | 105 | -2.634 | 0.228 | -1.459 | STRAND |
| PRO | 106 | 0 | 0 | 0 |  |
| ALA | 107 | 2.01 | -0.82 | 1.479 | HELIX |
| LYS+ | 108 | 0.453 | -1.153 | 2.645 | HELIX |
| MET | 109 | 3.61 | 0.111 | 3.014 | HELIX |
| ALA | 110 | 2.473 | -1.465 | 2.99 | HELIX |
| GLU- | 111 | 1.81 | 0.278 | 2.313 | HELIX |
| LYS+ | 112 | -0.081 | -1.551 | 2.188 | HELIX |
| ALA | 113 | 2.29 | -1.271 | 2.67 | HELIX |
| LYS+ | 114 | 2.622 | -0.358 | 3.342 | HELIX |
| GLN | 115 | 2.489 | -0.997 | 3.908 | HELIX |
| ILE | 116 | 4.197 | -1.061 | 4.323 | HELIX |
| TYR | 117 | 3.734 | -0.49 | 3.828 | HELIX |
| GLU- | 118 | 1.339 | -0.663 | 2.318 | HELIX |
| GLU- | 119 | 1.433 | 0.704 | -0.202 |  |
| PHE | 120 | -0.111 | 3.226 | -0.976 | STRAND |
| ILE | 121 | -1.283 | -0.963 | -1.416 | STRAND |
| GLN | 122 | -1.148 | -0.556 | 0.821 |  |
| THR | 123 | 2.652 | -0.722 | 2.319 | HELIX |
| GLU- | 124 | 0.753 | -3.421 | 3.107 | HELIX |
| ALA | 125 | -0.191 | -1.963 | 2.489 | HELIX |
| PRO | 126 | 1.164 | -0.356 | 0.046 |  |
| LYS+ | 127 | -2.924 | 0.23 | -0.776 |  |
| GLU- | 128 | -0.391 | 0.304 | -2.563 | STRAND |
| VAL | 129 | -3.014 | 0.825 | -1.224 | STRAND |
| ASN | 130 | -0.622 | -1.484 | -2.14 | STRAND |
| ILE | 131 | -2.183 | 1.261 | -0.861 |  |
| ASP- | 132 | 0 | 0 | 0 |  |
| HIS | 133 | 0 | 0 | 0 |  |
| PHE | 134 | 2.752 | -1.855 | 2.934 | HELIX |
| THR | 135 | 4.195 | 0 | 4.706 | HELIX |
| LYS+ | 136 | 4.673 | -0.643 | 4.16 | HELIX |
| ASP- | 137 | 3.219 | 0.25 | 3.747 | HELIX |
| ILE | 138 | 1.65 | -1.307 | 4.007 | HELIX |
| THR | 139 | 6.095 | 0 | 5.031 | HELIX |
| MET | 140 | 3.276 | -2.765 | 5.244 | HELIX |
| LYS+ | 141 | 2.506 | -1.09 | 3.64 | HELIX |
| ASN | 142 | 0.343 | -0.939 | 1.633 | HELIX |
| LEU | 143 | -0.845 | -0.867 | 2.105 | HELIX |
| VAL | 144 | 3.659 | -1.353 | 0.793 |  |
| GLU- | 145 | -2.408 | 0.248 | 0.848 |  |
| PRO | 146 | 0.347 | 0.158 | -2.531 |  |
| SER | 147 | -1.745 | 3.38 | -0.625 |  |
| LEU | 148 | 2.512 | -0.548 | -1.149 | STRAND |
| SER | 149 | -1.026 | 0.355 | 2.193 | HELIX |
| SER | 150 | 5.483 | 0.582 | 1.331 | HELIX |
| PHE | 151 | 0.302 | -0.172 | 3.5 | HELIX |
| ASP- | 152 | 4.024 | -1.102 | 3.321 | HELIX |
| MET | 153 | 3.039 | -1.323 | 4.271 | HELIX |
| ALA | 154 | 2.283 | -1.043 | 4.317 | HELIX |
| GLN | 155 | 3.674 | -1.589 | 3.857 | HELIX |
| LYS+ | 156 | 2.458 | -0.524 | 3.712 | HELIX |
| ARG+ | 157 | 1.299 | -1.592 | 1.926 | HELIX |
| ILE | 158 | -0.096 | 0 | 2.163 | HELIX |
| HIS | 159 | 5.141 | 1.447 | 2.201 | HELIX |
| ALA | 160 | 1.893 | -1.112 | 3.144 | HELIX |
| LEU | 161 | 2.635 | -0.097 | 2.349 | HELIX |
| MET | 162 | 3.973 | 2.664 | 2.141 | HELIX |
| GLU- | 163 | 2.587 | 0.206 | 1.425 | HELIX |
| LYS+ | 164 | 0.916 | 0.332 | 0.787 |  |
| ASP- | 165 | 1.038 | 1.642 | 1.26 | HELIX |
| SER | 166 | 3.514 | -0.286 | 3.718 | HELIX |
| LEU | 167 | 6.324 | -1.635 | 5.233 | HELIX |
| PRO | 168 | 2.832 | -1.109 | 4.752 |  |
| ARG+ | 169 | 2.418 | 0.061 | 2.974 | HELIX |
| PHE | 170 | 1.425 | -1.2 | 2.779 | HELIX |
| VAL | 171 | 1.197 | -2.157 | 1.716 | HELIX |
| ARG+ | 172 | -0.949 | -0.117 | -0.147 |  |
| SER | 173 | -0.084 | 2.879 | -0.422 |  |
| GLU- | 174 | 1.553 | -0.975 | 0.821 |  |
| PHE | 175 | 2.312 | -0.587 | 2.797 | HELIX |
| TYR | 176 | 2.312 | -0.653 | 2.821 | HELIX |
| GLN | 177 | 1.429 | -1.169 | 2.301 | HELIX |
| GLU- | 178 | 0.765 | -0.575 | 1.657 | HELIX |
| LEU | 179 | 0.679 | -0.353 | 0.727 |  |
| ILE | 180 | -0.941 | -0.749 | 0.496 |  |
| LYS+ | 181 | 1.188 | 0.539 | 0.229 |  |

**FIGURES**


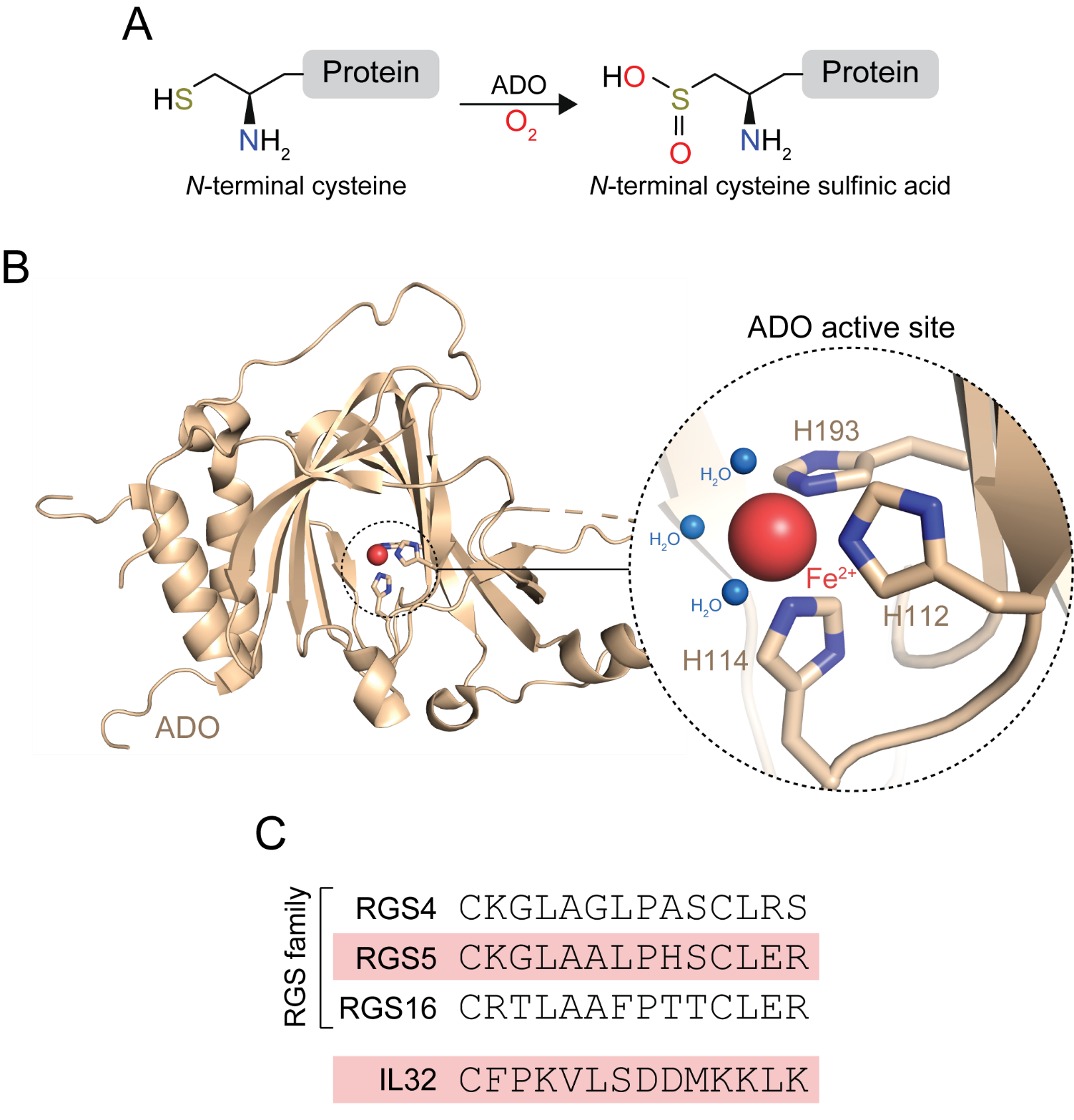


**Figure S1. Overview of 2-aminoethanethiol (cysteamine) dioxygenase (ADO) biochemistry and structure. A.** ADO utilises both atoms of O_2_ (red) to catalyse the sulfinylation of the *N*t-cys residue of its protein substrates. This reaction is inactivated in hypoxia. **B.** The X-ray crystal structure of ADO (PDB ID: 7REI) (1). *Left*: Top-down view into the β-barrel structure of ADO (*wheat*) that hosts the proteins active site (centre marked by a *dashed circle*). *Right inset*: A close-up of ADOs active site. The triad of histidine residues that coordinate the proteins iron cofactor (*coral sphere*) are displayed as sticks. The three water molecules that occupy the remainder of the metal ligation sites when the protein is at rest are represented as *blue spheres*. **C.** Sequence alignment of *N*-terminal 14 residues of ADOs known protein targets. The protein targets analysed in this study (RGS5 and IL32) are boxed in pink.


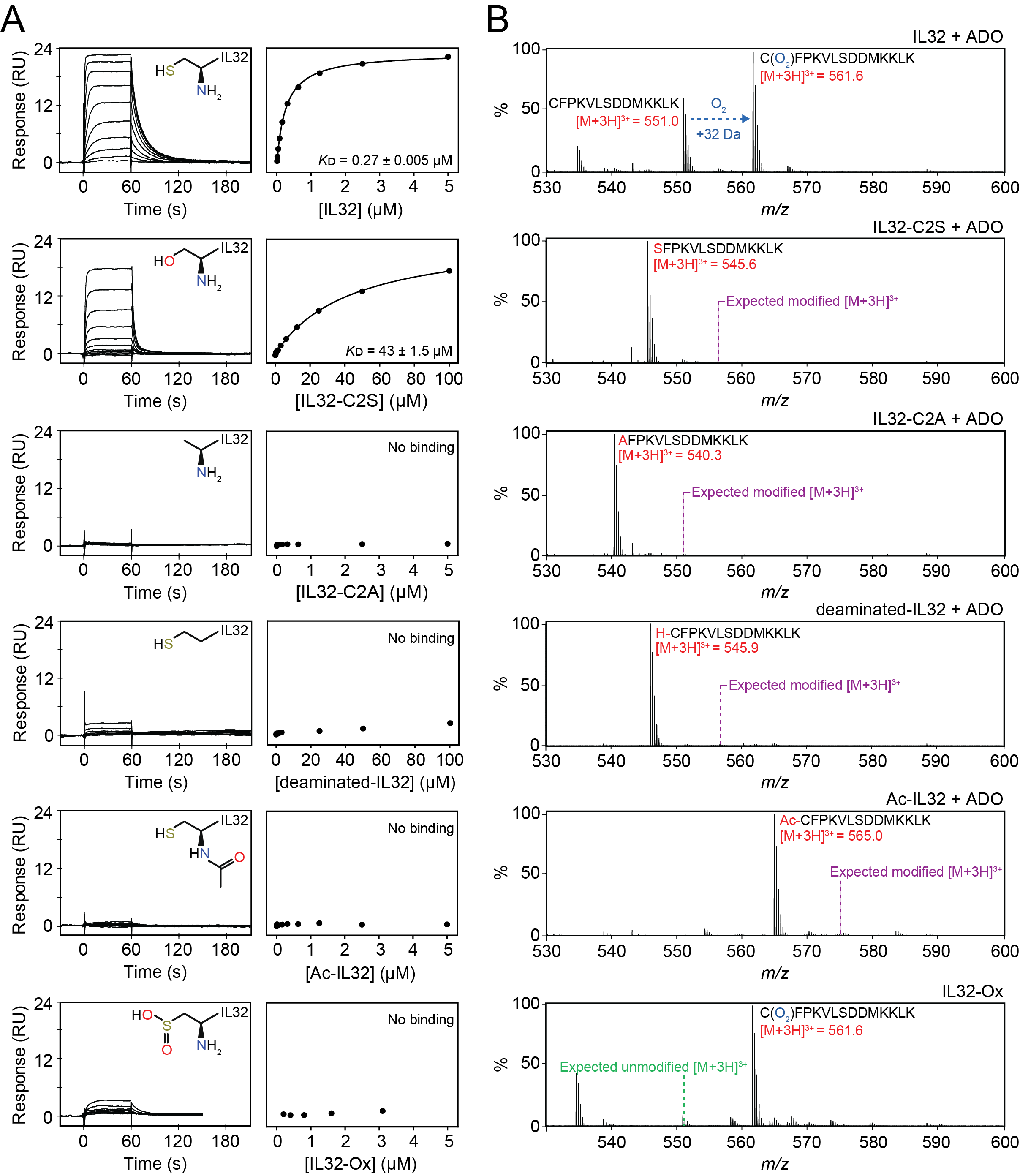


**Figure S2. ADO requires an unmodified *N*t-cys to enable an interaction with IL32. A.** *Left*: Representative SPR sensorgrams for the titrations of modified *N*t-cys IL32 peptides with ADO. *Right*: Fits of the equilibrium responses from the sensorgrams in the *left panels* to a 1:1 binding model. The identity, chemical structures of the modification state of the *N*t-cys of each peptide, and *K*ᴅ values are shown (*K*ᴅ given as the geometric mean of a minimum of three independent SPR measurements). **B.** LC-MS spectra showing the IL32 peptide species detected following a 45 second incubation of the modified *N*t-cys IL32 peptides (100 μM) with ADO (0.1 μM) at 37 °C. The expected +32 da mass shift resulting from sulfinylation of the native IL32 peptide in the presence of ADO is detected. The remaining IL32 peptides do not get sulfinylated by ADO. The positions of the peptide species expected upon oxidation (or unmodified IL32 in the case of the IL32-Ox peptide) by ADO are indicated.


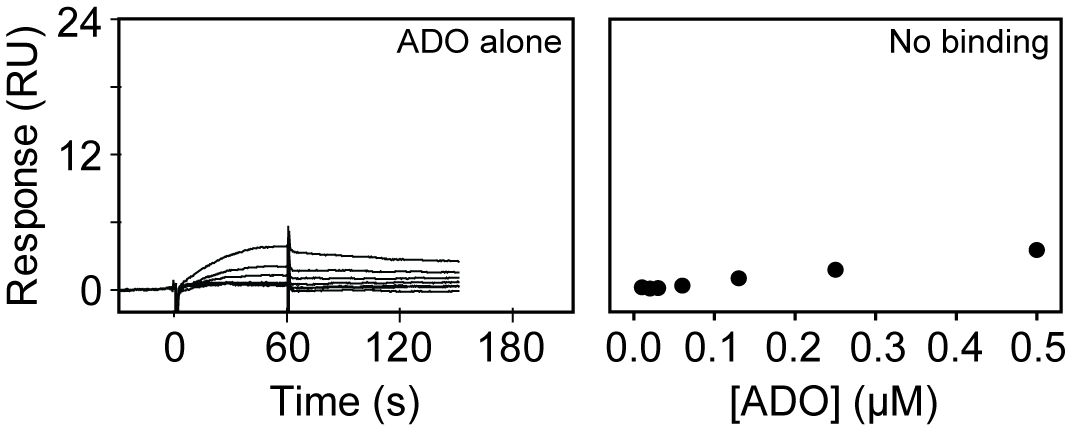


**Figure S3.** SPR sensorgrams (*left*) and plots of the equilibrium response points (*right*) obtained by titrating untagged ADO over the immobilised ADO surface used to measure interactions with modified *N*t-cys RGS5 (Fig. 1A) and IL32 (Fig. S2A) peptides. The same concentration of ADO that is present in/was used to prepare the RGS5-Ox and IL32-Ox peptide samples was used.


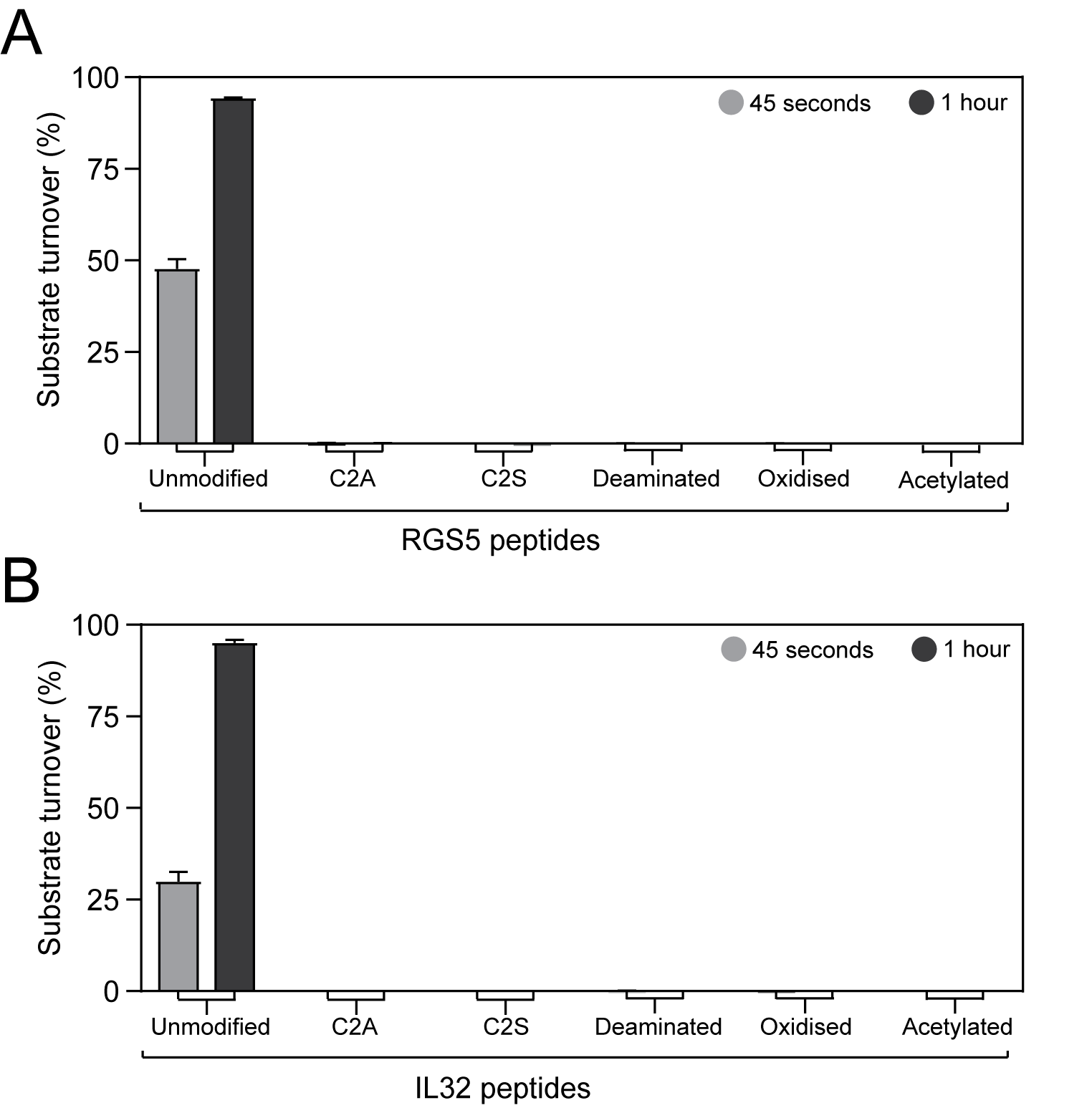


**Figure S4. The activity of ADO on modified *N*t-cys RGS5 and IL32 peptides at different time points.** Substrate turnover by ADO (0.1 μM) calculated by measuring the percentage of modified *N*t-cys RGS5 (**A**) and IL32 (**B**) peptide oxidation after 45 seconds and 1 hour (100 μM peptide, incubation at 37 °C) by LC-MS. Only the unmodified RGS5 and IL32 peptides can be sulfinylated by ADO. The average of three independent experiments are shown (error bars show the standard error).


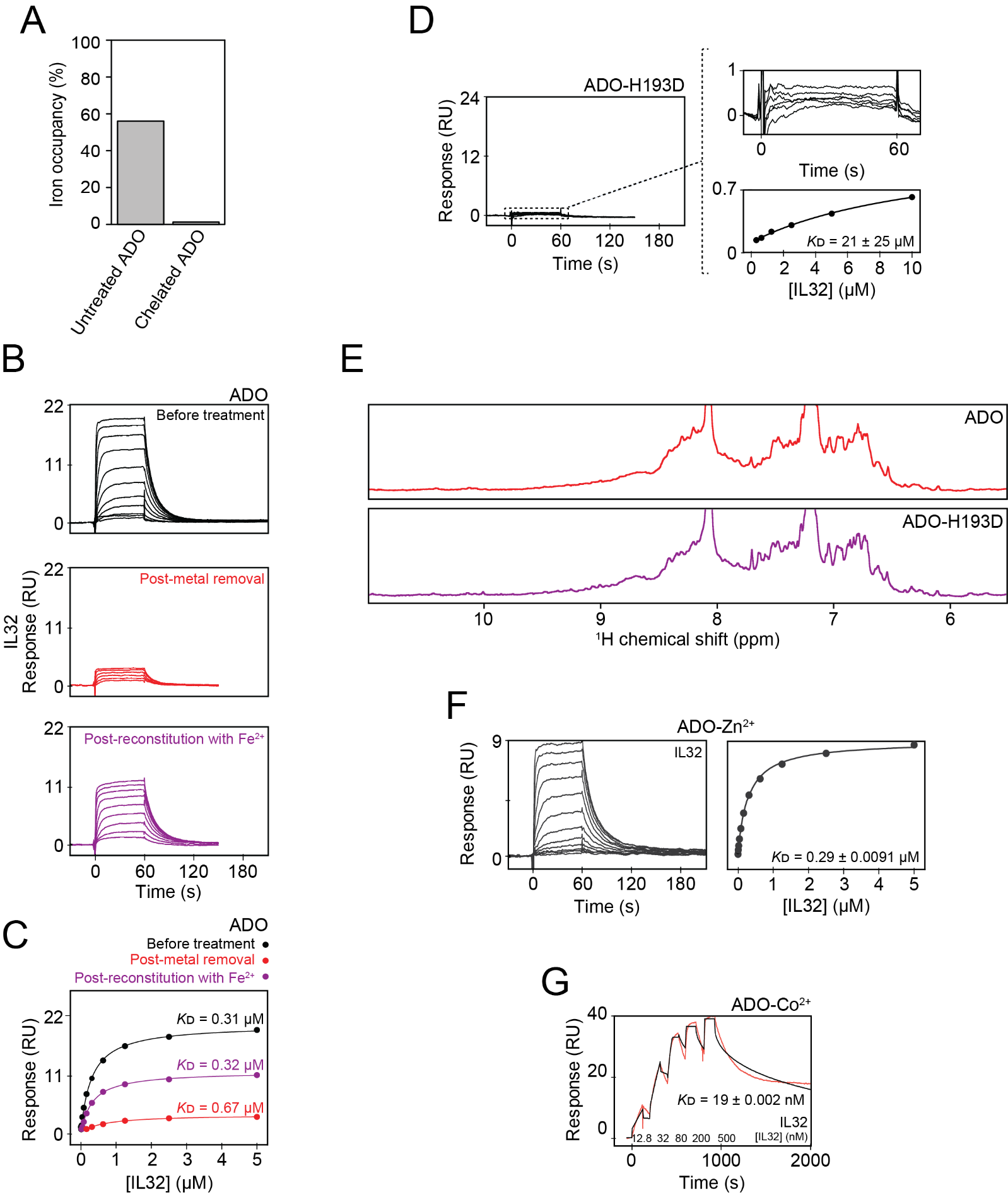


**Figure S5. ADO requires an active site metal for substrate binding. A.** The iron occupancy of the untreated ADO and metal chelated ADO determined by ICP-MS. The occupancy is provided as a percent based on amount of iron detected in the sample relative to the concentration of ADO present. The chelated ADO was prepared by including 10 mM 1, 10-phenanthroline and 100 mM EDTA during lysis and affinity chromatography when purifying the recombinant protein. **B.** Representative SPR sensorgrams for the titration of the native IL32 peptide with ADO before treatment (*top*), post-metal removal using 10 mM 1, 10-phenanthroline and 100 mM EDTA (*middle*), and post-reconstitution with iron using 0.1 mM FeSO­_4_ (supplemented with 12.5 mM sodium ascorbate) (*bottom*). **C.** Fits of the equilibrium responses from the sensorgrams in **A** to a 1:1 binding model. The *K*ᴅ values presented are calculated from the specific SPR data presented in this figure. **C.** *Left*: Representative SPR sensorgram for the titration of native IL32 peptide with ADO-H193. *Right*: Fits of the equilibrium responses from the sensorgrams in the *left panels* to a 1:1 binding model. The *K*ᴅ value is shown (*K*ᴅ given as the geometric mean of a minimum of three independent SPR measurements). **E.** One-dimensional 1D ^1^H-NMR spectra of native ADO and ADO-H192D. **F.** *Left*: Representative SPR sensorgram for the titration of the native IL32 peptide with ADO-Zn^2+^. *Right*: Fits of the equilibrium responses from the sensorgrams in the *left panels* to a 1:1 binding model. The *K*ᴅ value is shown (*K*ᴅ given as the geometric mean of a minimum of three independent SPR measurements). **G.** Representative single cycle kinetic (SCK) SPR sensorgram of the native IL32 peptide with ADO-Co^2+^. The sensorgram is shown in *red* and the fit to the data is shown in *black*. The concentrations of RGS5 used in the titration and the *K*ᴅ value is shown (*K*ᴅ given as the geometric mean of a minimum of three independent SPR measurements).


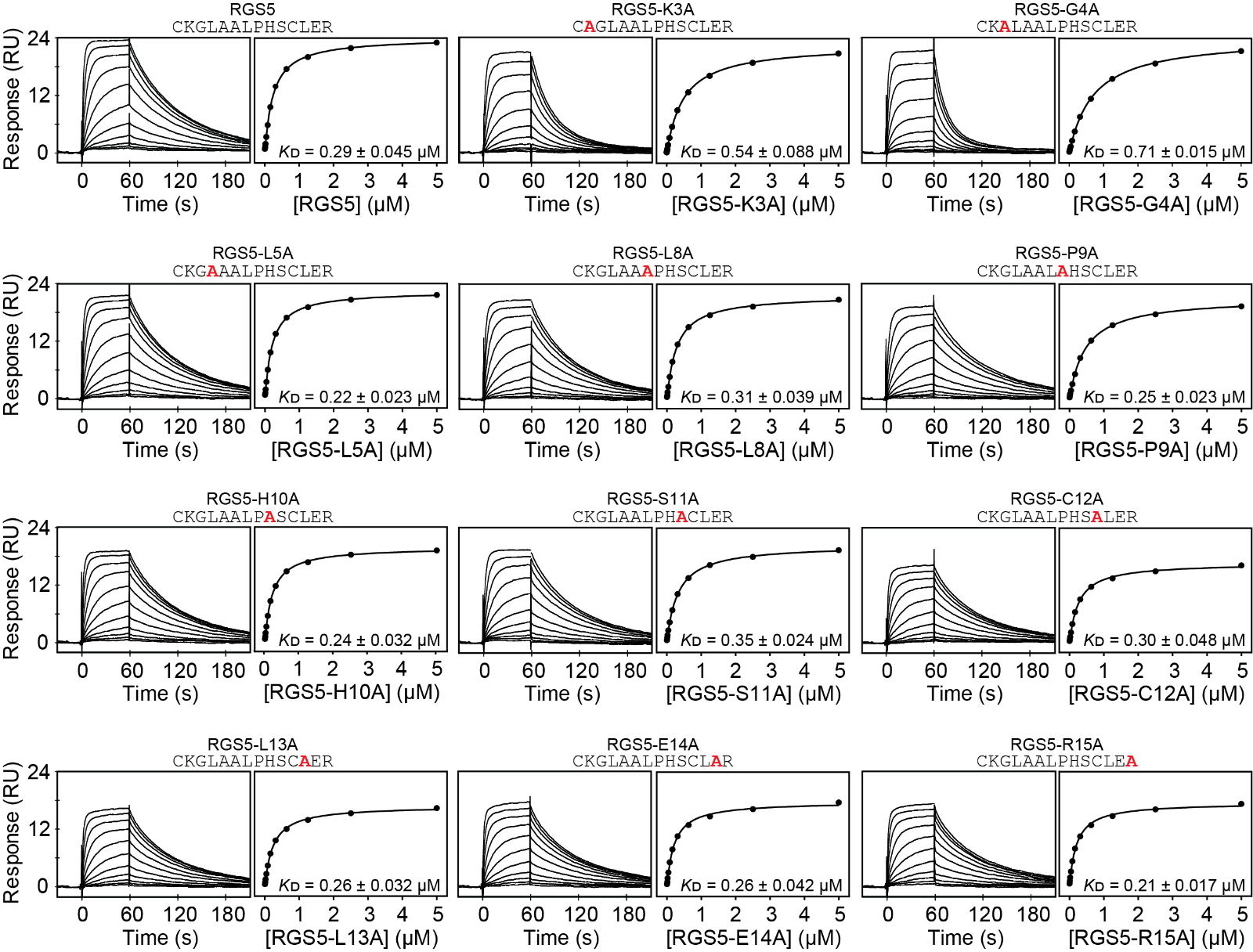


**Figure S6. SPR analysis of the RGS5 peptide alanine mutational scan.** The identity and sequence of each peptide is provided, and the alanine mutation is highlighted by *bold red lettering*. *Left*: Representative SPR sensorgram for the titration of given peptide with ADO. *Right*: Fits of the equilibrium responses from the sensorgrams in the *left panels* to a 1:1 binding model. The *K*ᴅ values are shown (*K*ᴅ given as the geometric mean of a minimum of three independent SPR measurements). Data for the native RGS5 peptide (first presented in Fig. 1A) and RGS5-K3A and RGS5-G4A (first presented in Fig. 3B) are provided for reference.


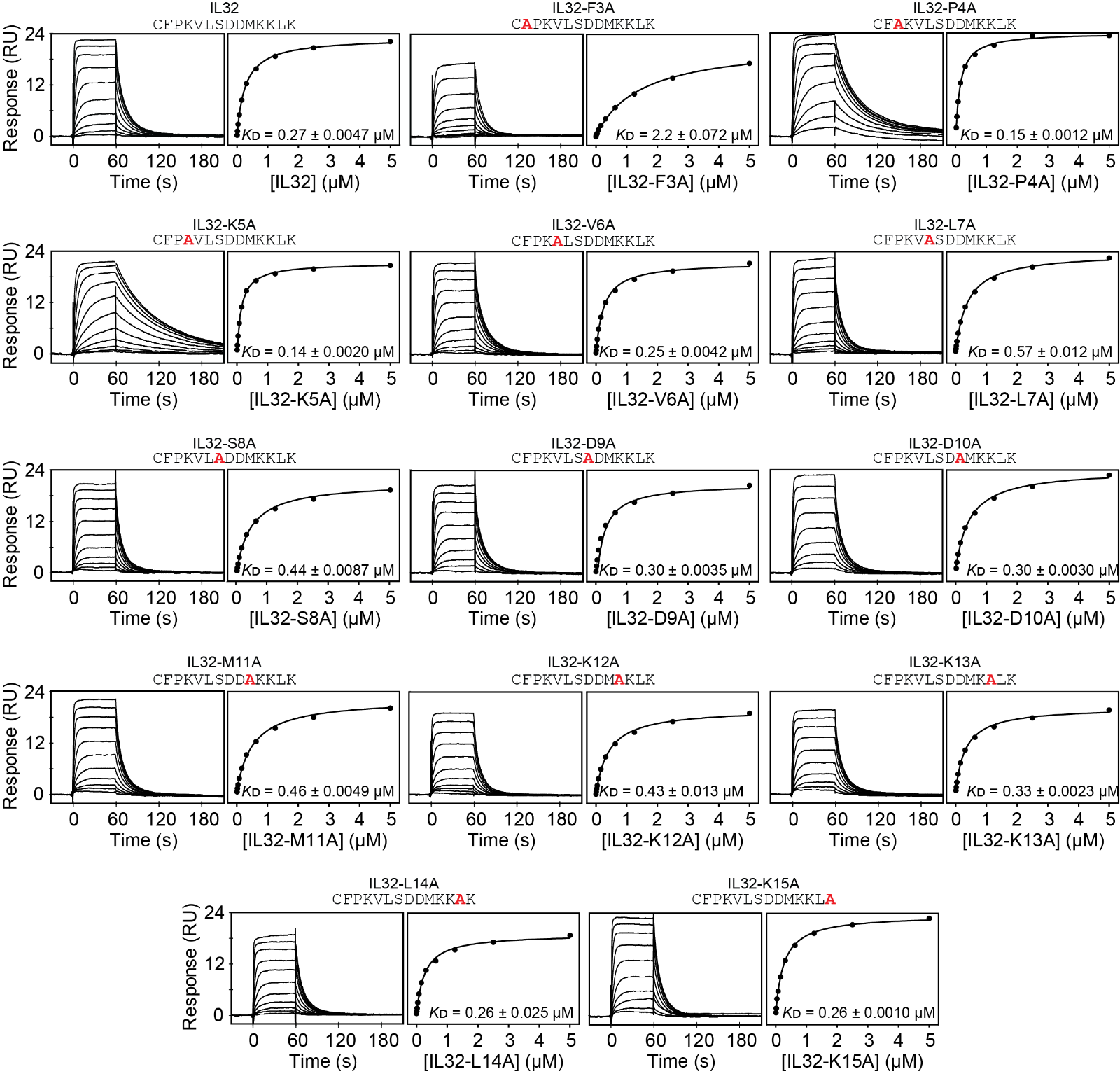


**Figure S7. SPR analysis of the IL32 peptide alanine mutational scan.** The identity and sequence of each peptide is provided, and the alanine mutation is highlighted by *bold red lettering*. *Left*: Representative SPR sensorgram for the titration of given peptide with ADO. *Right*: Fits of the equilibrium responses from the sensorgrams in the *left panels* to a 1:1 binding model. The *K*ᴅ values are shown (*K*ᴅ given as the geometric mean of a minimum of three independent SPR measurements). Data for the native IL32 peptide are provided for reference (first presented in Fig. S2A).


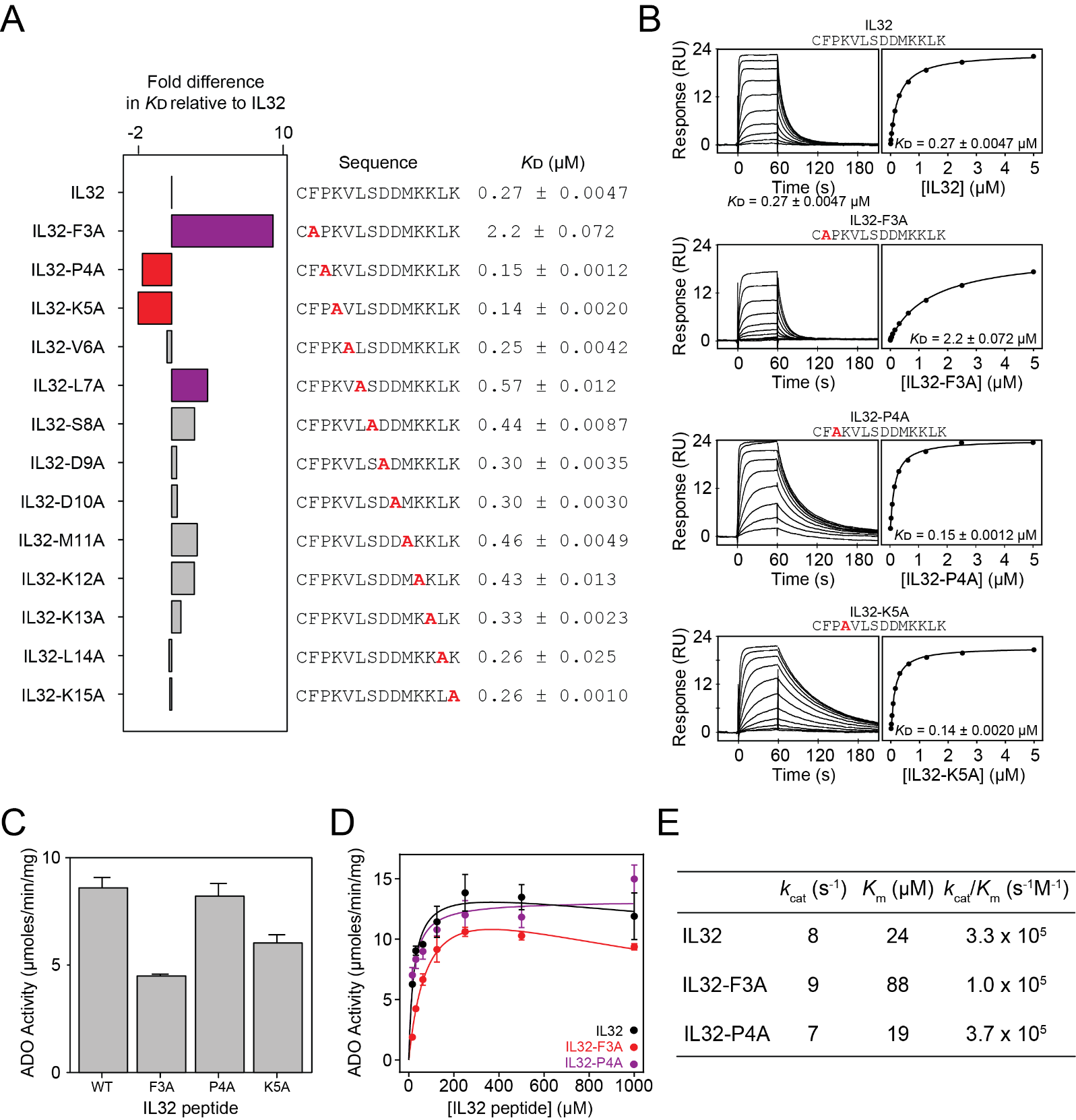


**Figure S8. SPR and enzyme kinetic analyses of the Il32 alanine mutational scan. A.** Fold different in *K*ᴅ relative to the native IL32 peptide. The substitutions that generate a greater than 1.5-fold reduction in *K*ᴅ are coloured in *purple*. The substitutions that have a greater than 1.5-fold increase in *K*ᴅ are coloured in *red*. The peptide sequences, with the mutation indicated in *bold red* lettering, and *K*ᴅ values for the interactions with ADO are provided (*K*ᴅ given as the geometric mean of a minimum of three independent SPR measurements). **B.** *Left*: Representative SPR sensorgram for the titration of native IL32, IL32-F3A, IL32-P4A, and IL32-K5A with ADO. *Right*: Fits of the equilibrium responses from the sensorgrams in the *left panels* to a 1:1 binding model. The *K*ᴅ value is shown (*K*ᴅ given as the geometric mean of a minimum of three independent SPR measurements). Data for the native IL32 peptide (first presented in Fig. S2A) and IL32-F3A, IL32-P4A, and IL32-K5A (first presented in Fig. S7) are provided for reference. **C.** The specific activity of ADO (0.05 μM) calculated by measuring the rate of the alanine substitution of the three amino acids that follow the *N*t-cys of IL32 by LC-MS (100 μM peptide, 45 second incubation at 37 °C). **D.** Michaelis-Menten kinetic plots for native IL32, IL32-F3A, and IL32-P4A performed in aerobic conditions at 37 °C. The average of three independent experiments are shown (error bars show the standard error). **E.** Table of the reaction kinetics for ADO catalysis of native IL32, IL32-F3A, and IL32-P4A calculated from the data presented in **D**.

**
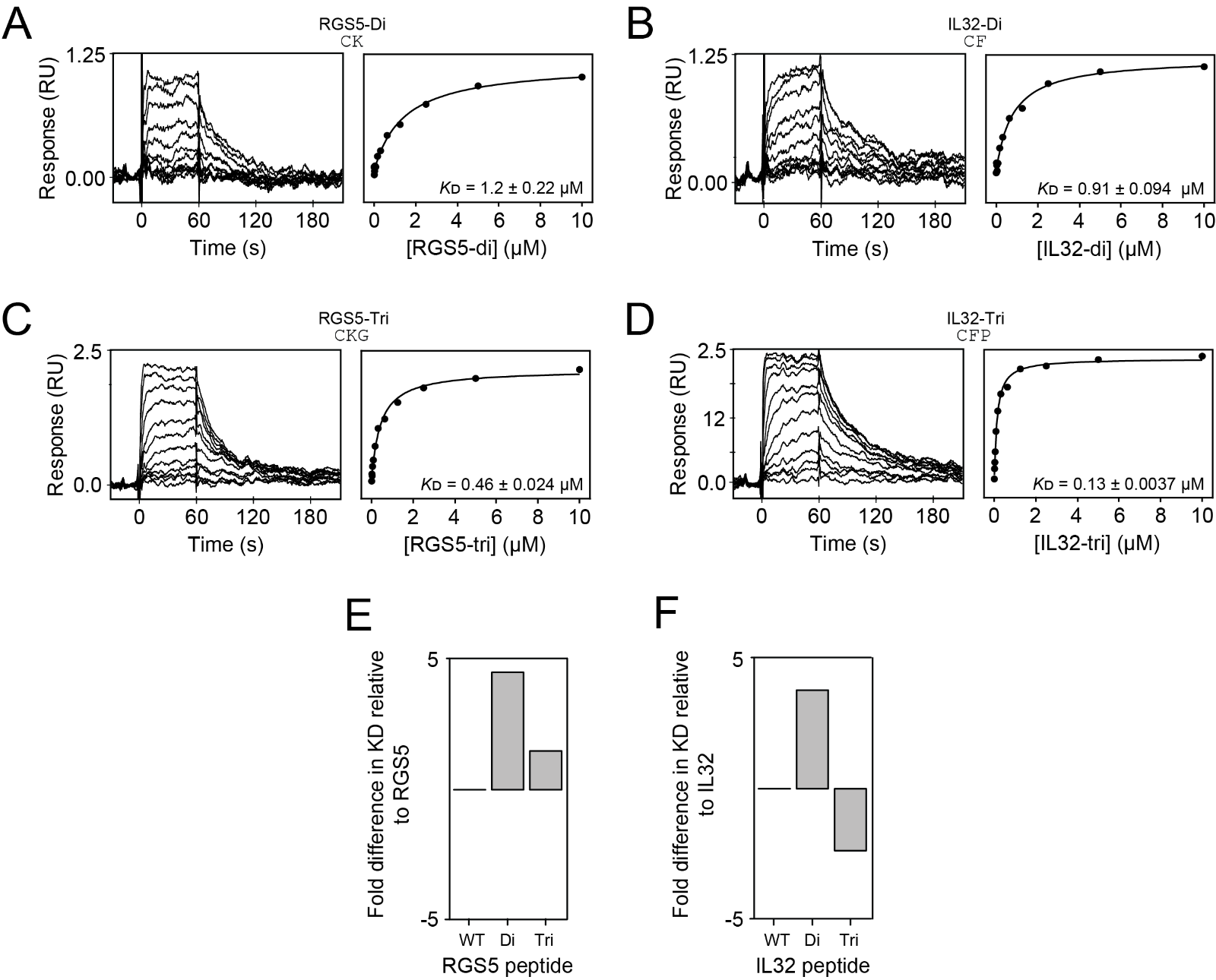
**

**Figure S9. SPR binding analyses of the interaction between short RGS5 and IL32 peptides and ADO.** SPR experiments for the titration of RGS5 dipeptide (**A**), IL32 dipeptide (**B**), RGS5 tripeptide (**C**), and IL32 tripeptide (**D**). Representative sensorgrams (*left*) and fits of the equilibrium responses from the sensorgrams in the to a 1:1 binding model (*right*) are presented. The *K*ᴅ values are shown (*K*ᴅ given as the geometric mean of a minimum of three independent SPR measurements). **E.** Fold different in *K*ᴅ of the short RGS5 peptides relative to the native RGS5 14 residue long peptide. **F.** Fold different in *K*ᴅ of the short IL32 peptides relative to the native IL32 14 residue long peptide.

**
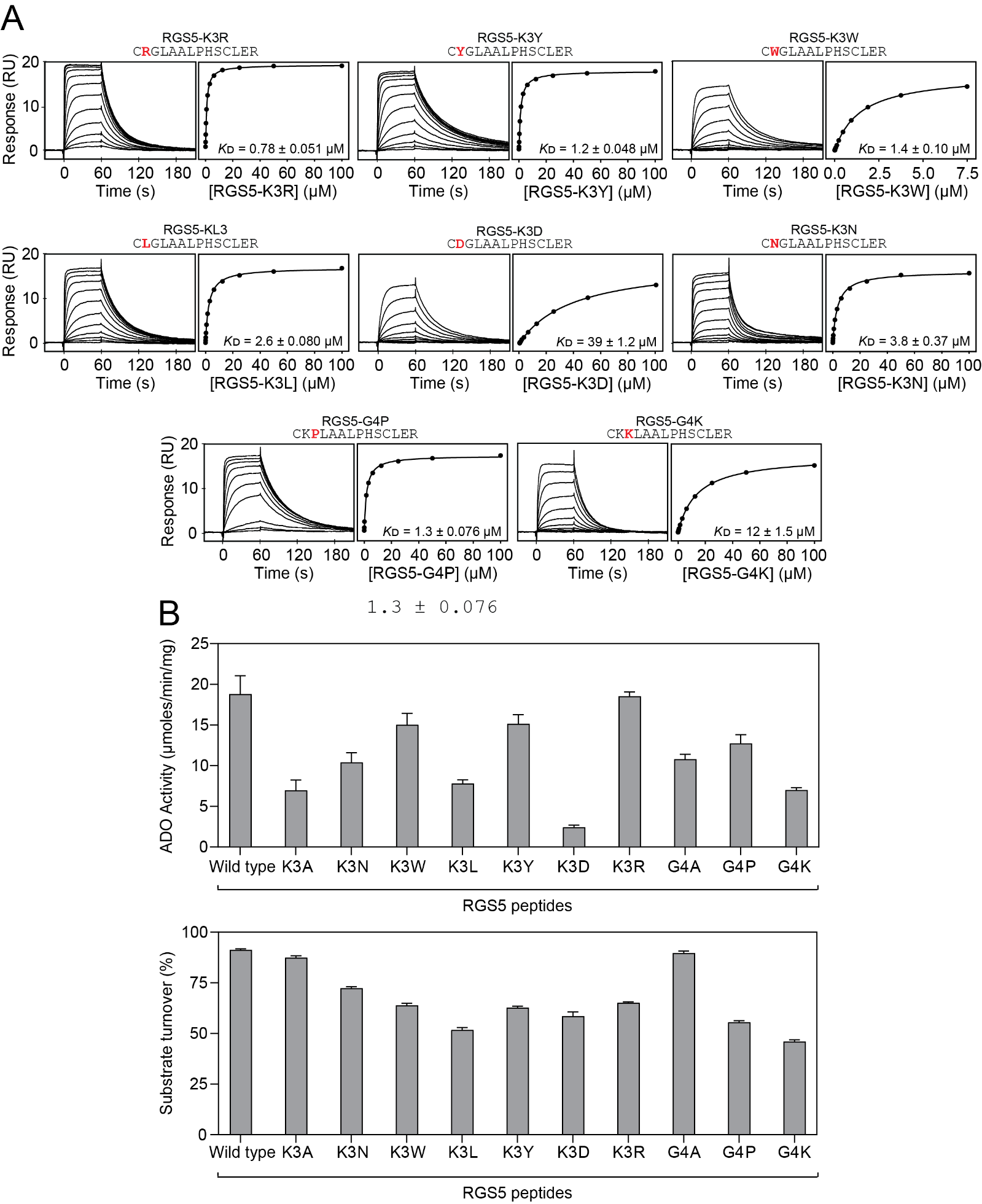
**

**Figure S10. SPR and enzyme kinetic analyses of the RGS5 peptide third and fourth position mutational scan. A.** The identity and sequence of each peptide is provided, and the mutation is highlighted by *bold red lettering*. *Left*: Representative SPR sensorgram for the titration of given peptide with ADO. *Right*: Fits of the equilibrium responses from the sensorgrams in the *left panels* to a 1:1 binding model. The *K*ᴅ values are shown (*K*ᴅ given as the geometric mean of a minimum of three independent SPR measurements). Data for the native RGS5 peptide is provided for reference. **B.** *Top graph*: The specific activity of ADO (0.1 μM) calculated by measuring the rate of the RGS5 peptide mutants by LC-MS (100 μM peptide, 45 second incubation at 37 °C). *Bottom graph*: Substrate turnover by ADO (0.1 μM) calculated by measuring the percentage of the RGS5 peptide mutant oxidation after 1 hour (100 μM peptide, incubation at 37 °C) by LC-MS.

**
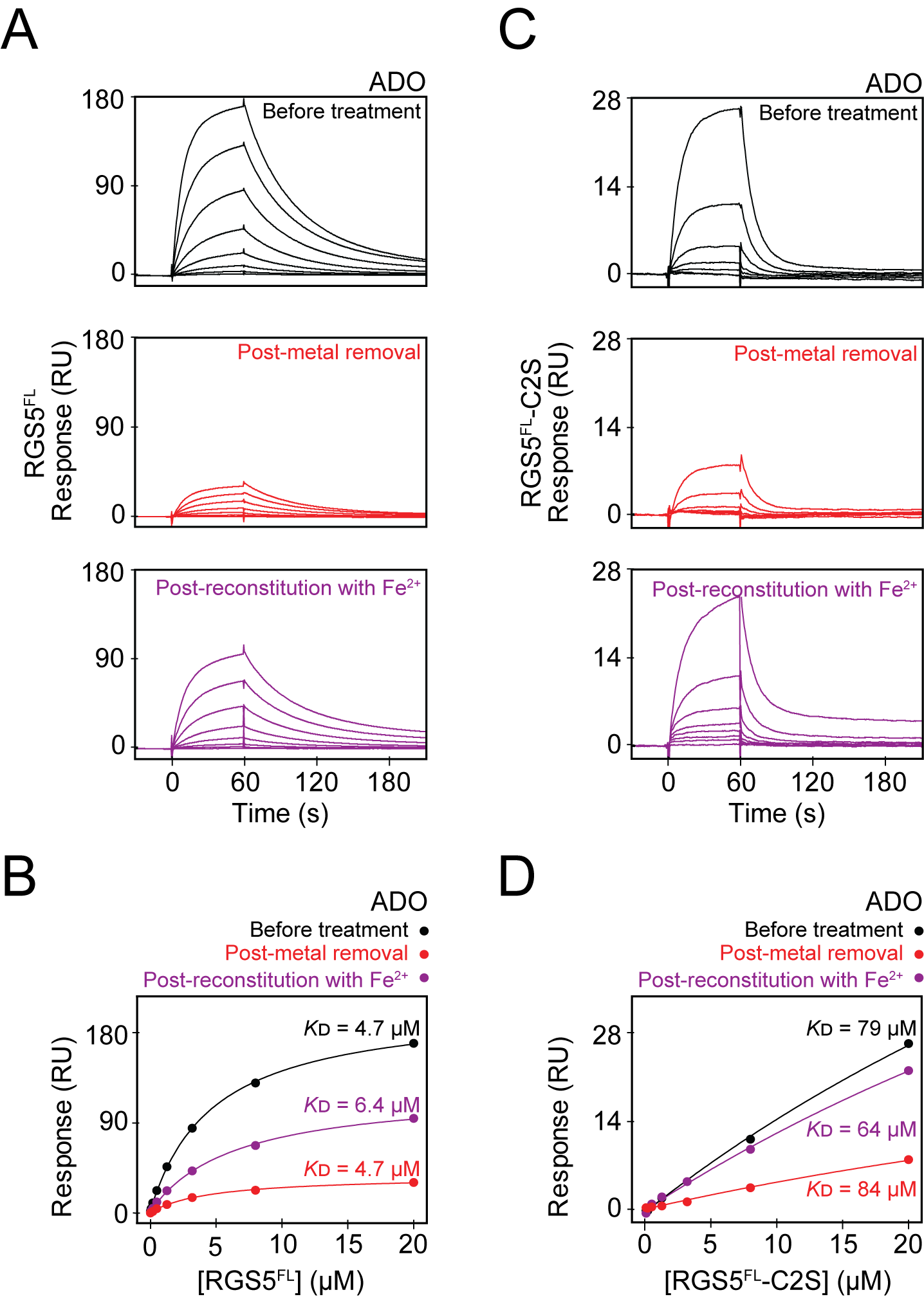
**

**Figure S11. Metal chelation SPR analysis of the interaction between ADO and full-length RGS5 (RGS5^FL^). A.** Representative SPR sensorgrams for the titration of RGS5^FL^ with ADO before treatment (*top*), post-metal removal using 10 mM 1, 10-phenanthroline and 100 mM EDTA (*middle*), and post-reconstitution with iron using 0.1 mM FeSO­_4_ (supplemented with 12.5 mM sodium ascorbate) (*bottom*). **B.** Fits of the equilibrium responses from the sensorgrams in **A** to a 1:1 binding model. The *K*ᴅ values presented are calculated from the specific SPR data presented in this figure. **C.** Representative SPR sensorgrams for the titration of RGS5^FL^-C2S with ADO before treatment (*top*), post-metal removal using 10 mM 1, 10-phenanthroline and 100 mM EDTA (*middle*), and post-reconstitution with iron using 0.1 mM FeSO­_4_ (supplemented with 12.5 mM sodium ascorbate) (*bottom*). **D.** Fits of the equilibrium responses from the sensorgrams in **C** to a 1:1 binding model. The *K*ᴅ values presented are calculated from the specific SPR data presented in this figure.

**
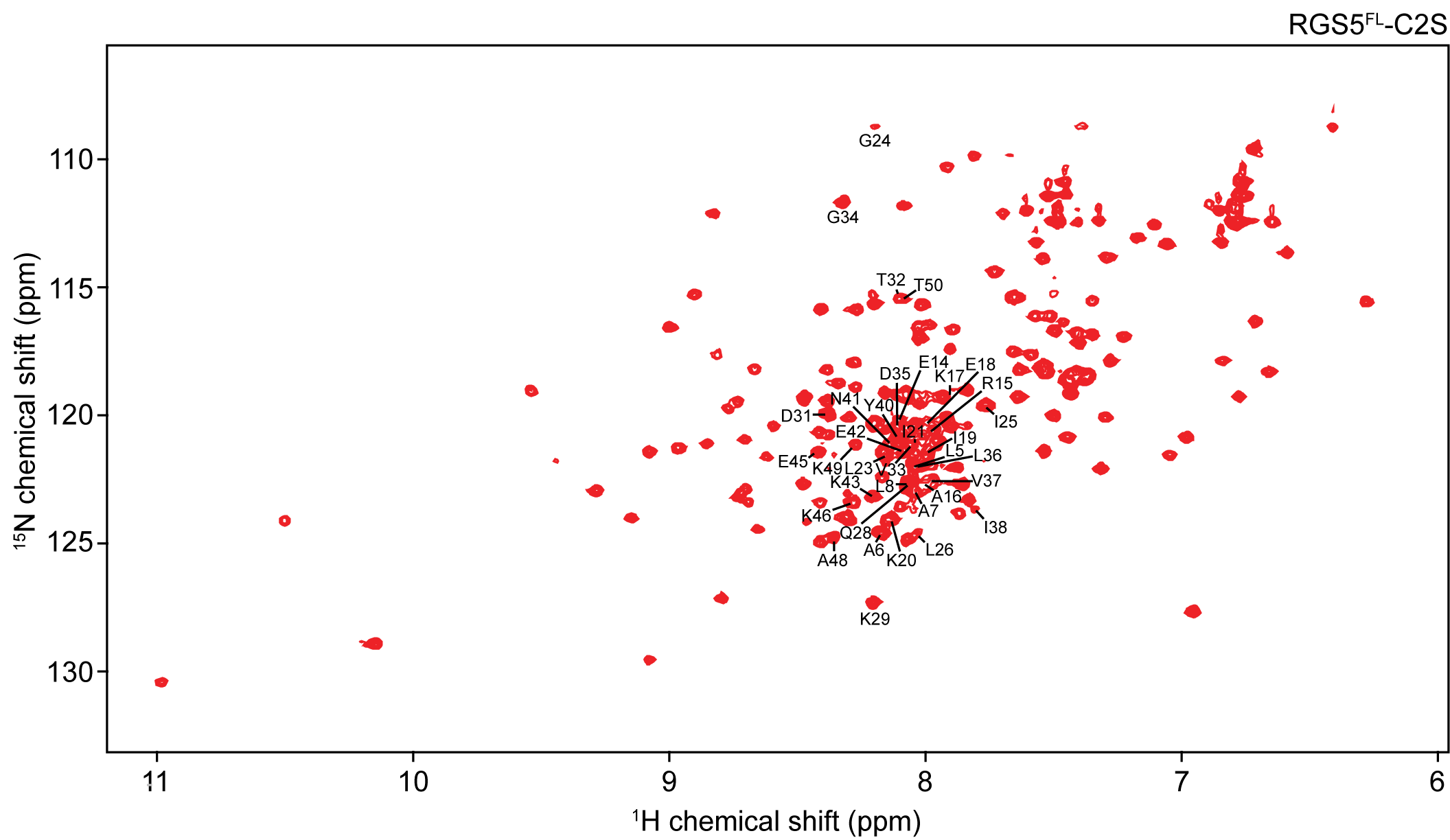
**

**Figure S12. Two-dimensional ^15^N-HSQC NMR spectrum of RGS5^FL^-C2S.** The positions of the chemical shifts corresponding to the disordered region of RGS5^FL^-C2S are labelled.


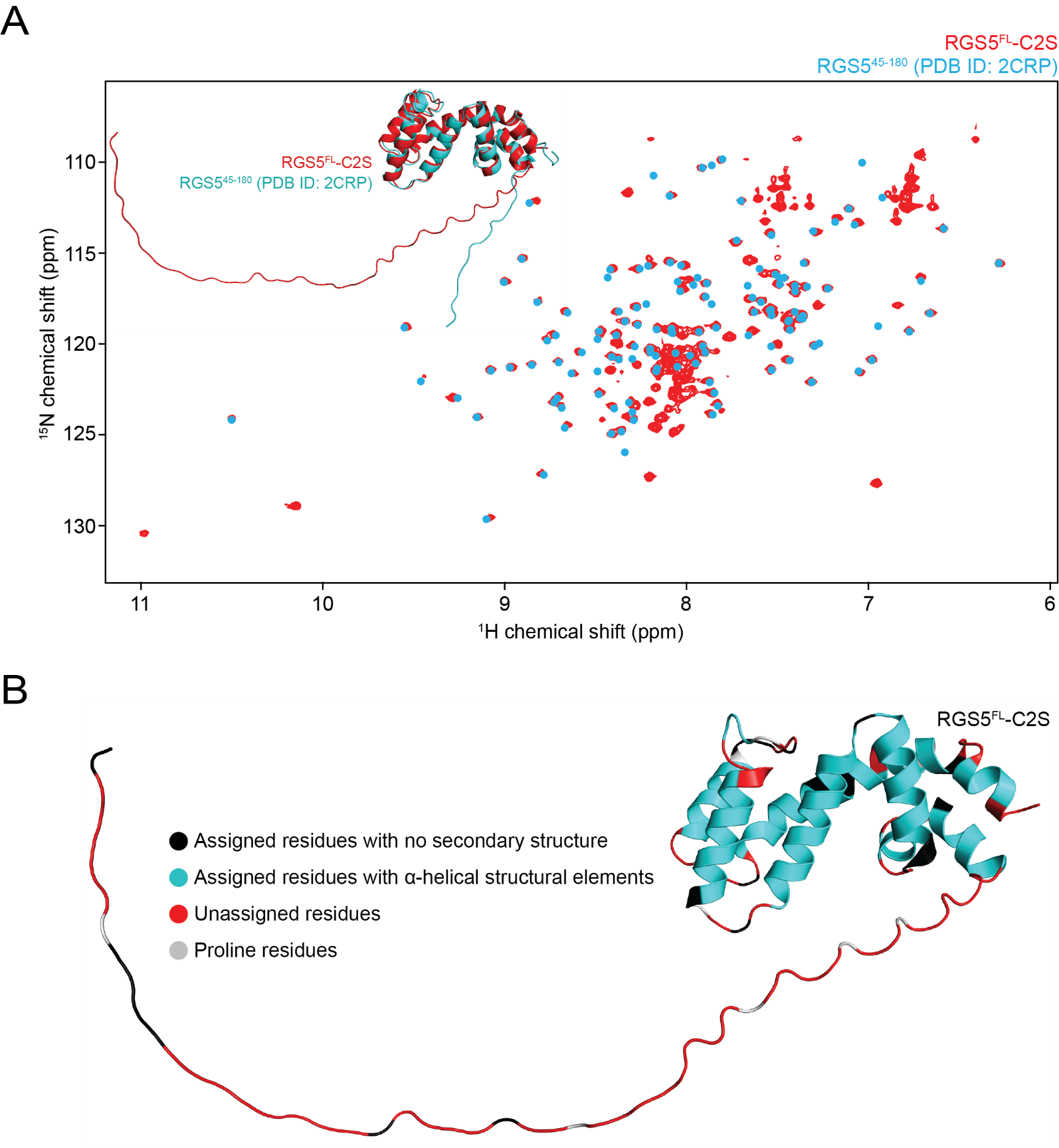


**Figure S13. Secondary structure analysis of RGS5^FL^-C2S. A.** Overlay of the ^1^H and ^15^N chemical shifts of the globular folded domain of RGS5 (PDB ID of the solution structure: 2CRP) with the ^15^N-HSQC of RGS5^FL^-C2S generated for this study. An overlay of the solution structure of the globular domain and the AlphaFold prediction of full-length RGS5 is shown in an inset (2). The chemical shifts for the globular domain of RGS5 used to solve the solution structure were provided by the Yokoyama Laboratory based at RIKEN (Japan). **B.** Secondary structure analysis of RGS5^FL^-C2S, generated from calculating the random coil deviation for CA and CB shifts of RGS5^FL^-CS2 (provided in Table S7), demonstrates that the *N*-terminal tail of RGS5 is disordered in solution.


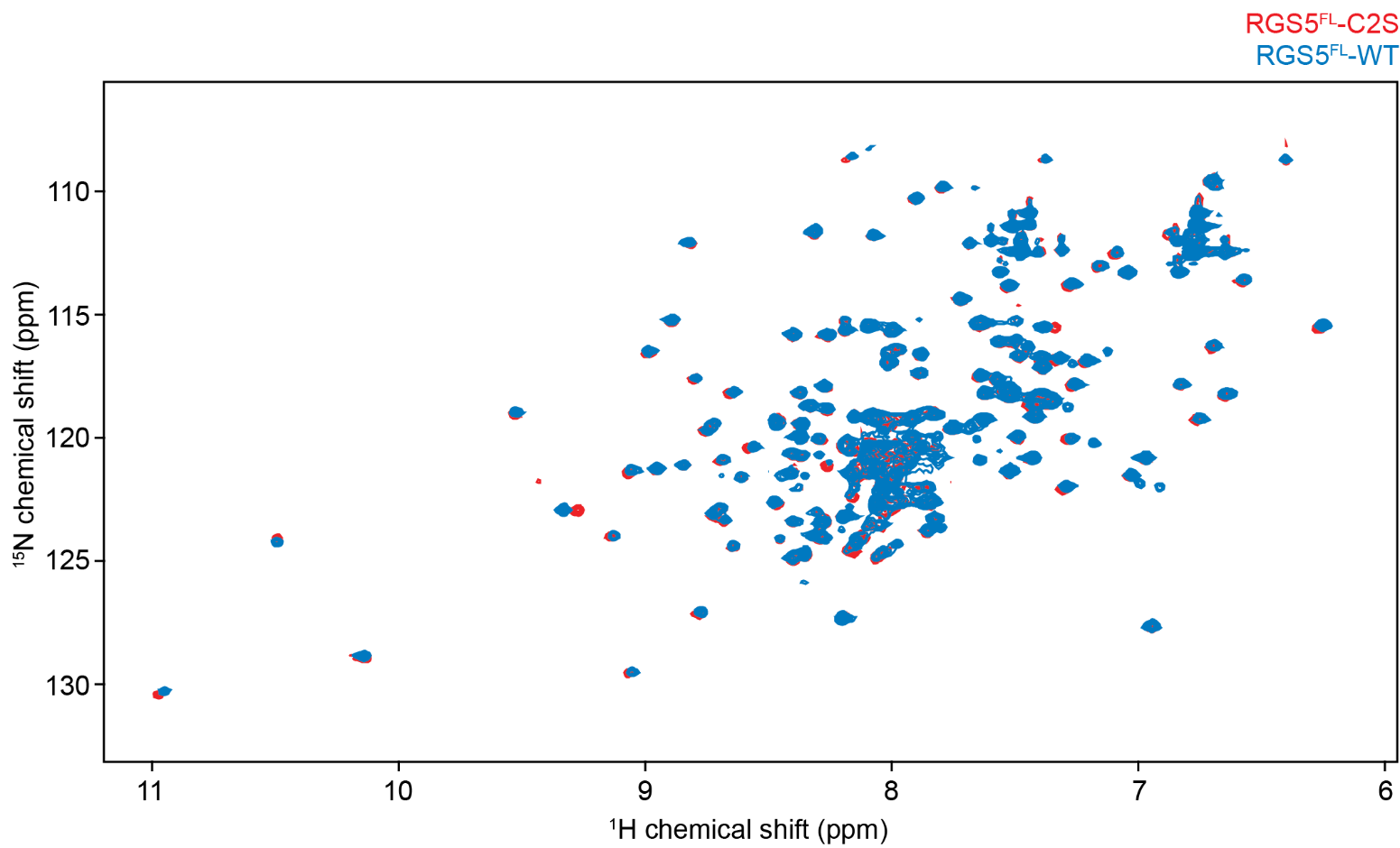


**Figure S14.** Overlay of the ^15^N-HSQC NMR spectra of RGS5^FL^ and RGS5^FL^-C2S.


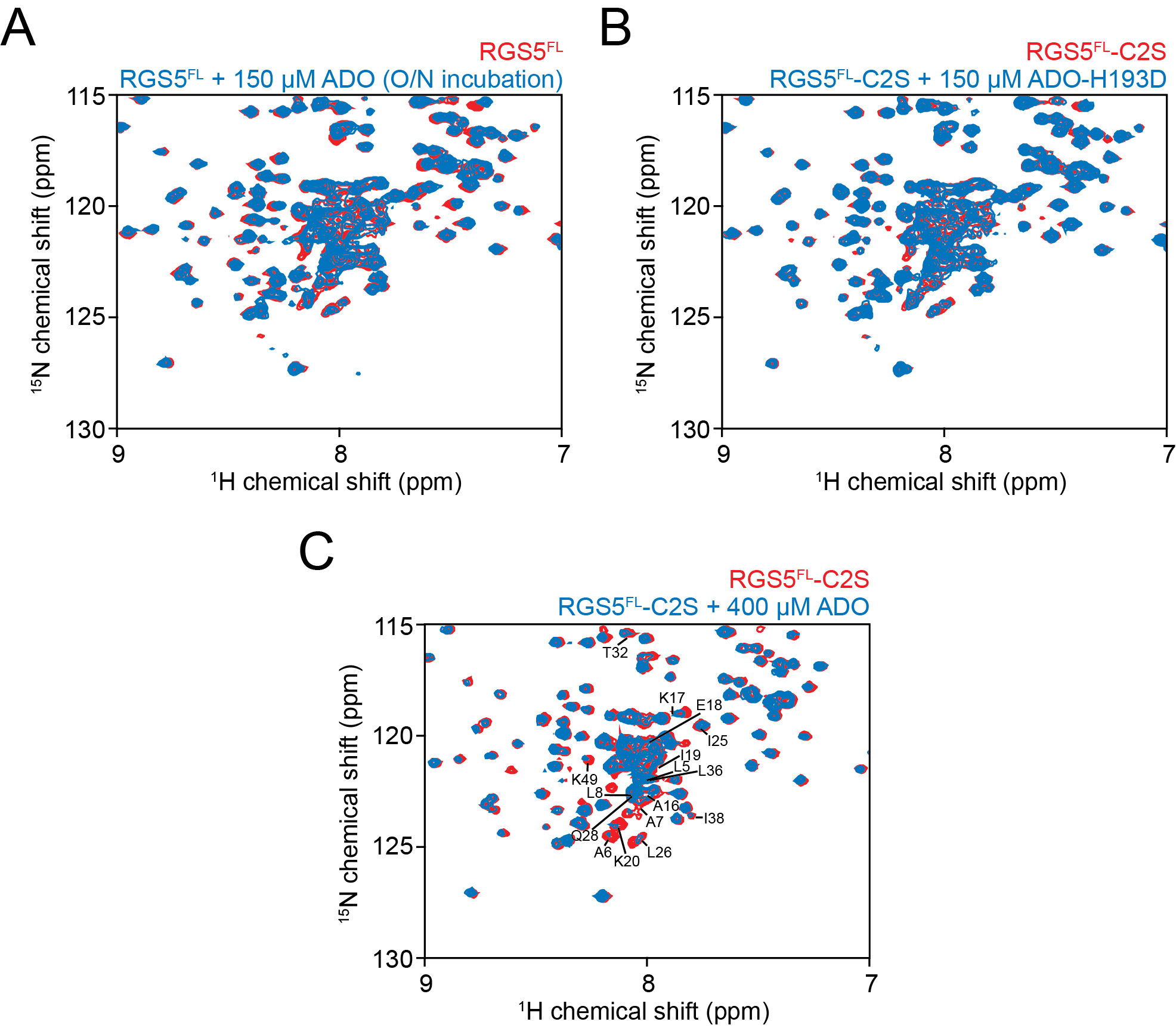


**Figure S15. ^15^N-HSQC titrations of ADO and full-length RGS5.** For all titrations, RGS5 was used at a concentration of 50 μM. **A.** ^15^N-HSQC spectra of RGS5^FL^ alone (*red*) and in the presence of 150 μM ADO (*blue*). The RGS5^FL^ + 150 μM ADO titration was collected after an overnight incubation step at room temperature. **B.** ^15^N-HSQC spectra of RGS5^FL^-C2S alone (*red*) and in the presence of 150 μM ADO-H193D (*blue*). C. ^15^N-HSQC spectra of RGS5^FL^-C2S alone (*red*) and in the presence of 400 μM ADO (*blue*). The assignments for some signals are indicated.

**REFERENCES**

1. Wang, Y., Shin, I., Li, J., and Liu, A. (2021) Crystal structure of human cysteamine dioxygenase provides a structural rationale for its function as an oxygen sensor. Journal of Biological Chemistry. 10.1016/j.jbc.2021.101176

2. Jumper, J., Evans, R., Pritzel, A., Green, T., Figurnov, M., Ronneberger, O., Tunyasuvunakool, K., Bates, R., Žídek, A., Potapenko, A., Bridgland, A., Meyer, C., Kohl, S. A. A., Ballard, A. J., Cowie, A., Romera-Paredes, B., Nikolov, S., Jain, R., Adler, J., Back, T., Petersen, S., Reiman, D., Clancy, E., Zielinski, M., Steinegger, M., Pacholska, M., Berghammer, T., Bodenstein, S., Silver, D., Vinyals, O., Senior, A. W., Kavukcuoglu, K., Kohli, P., and Hassabis, D. (2021) Highly accurate protein structure prediction with AlphaFold. Nature 2021 596:7873. 596, 583–589
